# Surface Modifications of High-Performance Polymer Polyetheretherketone (PEEK) to Improve Its Biological Performance in Dentistry

**DOI:** 10.3390/polym14245526

**Published:** 2022-12-16

**Authors:** Bidhari Pidhatika, Vania Tanda Widyaya, Prathima C. Nalam, Yogi Angga Swasono, Retno Ardhani

**Affiliations:** 1 Research Center for Polymer Technology, National Research and Innovation Agency, Republic of Indonesia PRTPL BRIN Indonesia, Serpong, Tangerang Selatan 15314, Indonesia; 2 Collaborative Research Center for Biomedical Scaffolds, National Research and Innovation Agency of the Republic Indonesia and Universitas Gadjah Mada, Jalan Denta No. 1, Sekip Utara, Yogyakarta 55281, Indonesia; 3 Department of Materials Design and Innovation, University at Buffalo, Buffalo, NY 14260-1900, USA; 4 Department of Dental Biomedical Science, Faculty of Dentistry, Universitas Gadjah Mada, Jalan Denta No. 1, Sekip Utara, Yogyakarta 55281, Indonesia

**Keywords:** osseointegration of PEEK, PEEK in dentistry, tribology of PEEK, polyetheretherketone, surface modifications, dental applications, bonding strength, surface topography, adhesive in dentistry

## Abstract

This comprehensive review focuses on polyetheretherketone (PEEK), a synthetic thermoplastic polymer, for applications in dentistry. As a high-performance polymer, PEEK is intrinsically robust yet biocompatible, making it an ideal substitute for titanium—the current gold standard in dentistry. PEEK, however, is also inert due to its low surface energy and brings challenges when employed in dentistry. Inert PEEK often falls short of achieving a few critical requirements of clinical dental materials, such as adhesiveness, osseoconductivity, antibacterial properties, and resistance to tribocorrosion. This study aims to review these properties and explore the various surface modification strategies that enhance the performance of PEEK. Literatures searches were conducted on Google Scholar, Research Gate, and PubMed databases using PEEK, polyetheretherketone, osseointegration of PEEK, PEEK in dentistry, tribology of PEEK, surface modifications, dental applications, bonding strength, surface topography, adhesive in dentistry, and dental implant as keywords. Literature on the topics of surface modification to increase adhesiveness, tribology, and osseointegration of PEEK were included in the review. The unavailability of full texts was considered when excluding literature. Surface modifications via chemical strategies (such as sulfonation, plasma treatment, UV treatment, surface coating, surface polymerization, etc.) and/or physical approaches (such as sandblasting, laser treatment, accelerated neutral atom beam, layer-by-layer assembly, particle leaching, etc.) discussed in the literature are summarized and compared. Further, approaches such as the incorporation of bioactive materials, e.g., osteogenic agents, antibacterial agents, etc., to enhance the abovementioned desired properties are explored. This review presents surface modification as a critical and essential approach to enhance the biological performance of PEEK in dentistry by retaining its mechanical robustness.

## 1. Introduction

In general, high-performance polymers are polymeric materials that can retain their desirable mechanical, thermal, and chemical properties under harsh environmental conditions, such as high temperature, high pressure, and corrosive chemicals [1]. In dentistry, high-performance polymers are utilized as resin composite and adhesive materials, prostheses or implant materials, etc. [2]. Polymers in dentistry include, for example, ester-free ether-based photo-CuAAC resin [3], poly(acrylic acid-co-itaconic acid) [4], epoxy resin [5], poly(etherketoneketone) (PEKK) [6], poly(etheretherketone) (PEEK) [6,7,8,9,10,11], poly(phenylene sulfone) (PPSU) [11], poly(methyl methacrylate) (PMMA) [12,13,14,15], poly(ethyl methacrylate) (PEMA) [13], and dimethacrylate (bis-acryl) polymers [13]. Further examples include amine-free methacrylate resins [16], copolymers composed of epigallocatechin-gallate (EGCG) methacrylate and triethylene glycol dimethacrylate (TEGDMA) [17], composite resins made of allyl(2-(2-(((allyloxy)carbonyl)oxy)benzoyl)-5-methoxyphenyl) carbonate (BZ-AL), Bisphenol-A glycidylmethacrylate (Bis-GMA) and silanized inorganic filler [18], BisGMA/TEGDMA resins [19,20,21], resin composites made of triethylene glycol divinylbenzyl ether (TEG-DVBE), urethane dimethacrylate (UDMA) [22], etc. Often, these polymers are combined with filler materials to improve their performance. For example, PMAA has been reinforced with zirconia and boron nitride nanopowders [14] and silicon nitride [15]; bisGMA/TEGDMA resins have been combined with either zinc oxide [20], nano-alumina [21], or methylaminopropyltrimethoxysilane-treated silica [19]; and bis-GMA oligomers have been reinforced with functionalized graphene and hydroxyapatite fillers [23]. 

**Figure 1:**
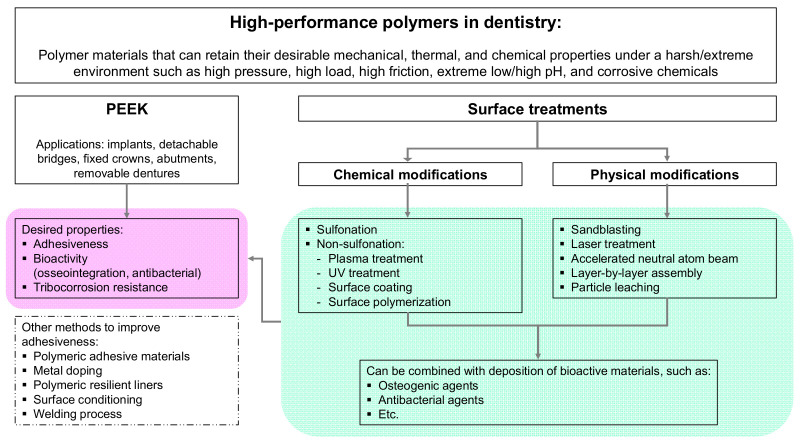
Discussion scope of this review article.

An excellent polymeric material for dental applications is PEEK, a high-temperature semi-crystalline, synthetic thermoplastic polymer [24]. Some literature reviews have been provided on the high-performance PEEK polymer in dentistry, and strategies for its modification already exist [6,25,26,27]. However, a comprehensive review that involves PEEK surface properties, PEEK surface modifications, and the corresponding performance of the PEEK or PEEK-containing composites that are highly related to dental applications is, to the best of the authors’ knowledge, not available. In particular, there is no review that includes the tribological properties of PEEK, as well as the surface modifications by surface polymerization, layer-by-layer assembly, and particle leaching methods. Thus, this review will focus on chemical and physical PEEK surface modifications in dentistry, and how these modifications affect the biological performance of PEEK, such as its adhesiveness (bonding strength), its bioactive properties (osseointegration, antibacterial properties), and its tribological properties, paying particular attention to the effects on the first two properties. The scope of this review is illustrated in Figure 1.

We applied the keywords PEEK, polyetheretherketone, osseointegration of PEEK, PEEK in dentistry, tribology of PEEK, surface modifications, dental applications, bonding strength, surface topography, adhesive in dentistry, and dental implant to Google Scholar, Research Gate, and PubMed database. Duplicate literature was sorted manually. Literature considered eligible for review if complied with inclusion criteria: English-written, with the topic on surface modification to increase adhesiveness, tribology, and osseointegration of PEEK. Pieces of literature were excluded if the full text was unavailable. Additional literature searching using keywords surface sulfonation of PEEK, PEEK plasma treatment, PEEK UV treatment, surface coating of PEEK, surface polymerization of PEEK, PEEK sandblasting, PEEK laser treatment, accelerated neutral atom beam PEEK, layer by layer PEEK, particle leaching of PEEK was performed. This step allowed us to do a thorough review of each strategy published in the literature.

## 2. High-Performance Polymer PEEK in Dentistry

Practically it is difficult to achieve all the desired properties for a given application using one single polymeric material, and PEEK is no exception. PEEK provides several exceptional properties that provide necessary reliability for dental materials, but it also suffers from other drawbacks, which, unless addressed, cannot be widely employed in the market. This section briefly explains the general attributes of PEEK and their impacts on dentistry.

### 2.1. The Superior Properties of PEEK

PEEK architecture consists of a linear aromatic backbone composed of functional ketone and ether groups [28]. PEEK has been claimed to exhibit low solubility and low water absorption and, at the same time, presents biocompatibility and compatibility with reinforcing materials. These properties enhance the fatigue-resistant, corrosion-resistant, radiolucent, stability at high temperatures, stability to sterilization processes, and ease of machinability [25,29,30,31]. PEEK has been widely used in dentistry to construct dental implants, implant abutments, fixed crowns, removable dentures, fixed and detachable bridges, etc. [32]. In the dental implant field, PEEK has been considered a strong candidate to substitute the gold standard, titanium, due to its superior esthetics and elastic modulus, which are comparable to the bulk properties of human bones [31,33]. While Ti and its alloys exhibit a high elastic modulus (110 GPa), PEEK has an elastic modulus (3–4 GPa) similar to human cortical bone (7–30 GPa) [34], preventing adverse stress-shielding effects [35,36]. Furthermore, PEEK is radiolucent, which is beneficial for monitoring osseointegration progress using imaging techniques such as X-rays. Ti-based dental materials tend to scatter X-rays hindering visualization of, for example, implant position and osseointegration progress [37].

Further, the studies also indicate the superior biocompatible nature of PEEK compared to Ti. For example, higher osteoblast viability and proliferation were detected on PEEK surfaces than on Ti6Al4V surfaces. When the PEEK was modified with 5% beta-tricalcium phosphate, an enhanced osteoblast differentiation was observed [38]. Peng et al. compared PEEK to the conventional materials Ti6Al4V and yttria-stabilized tetragonal zirconia polycrystal (Y-TZP) in terms of their cytotoxicity to human oral fibroblast. In vitro experiments showed the performance of PEEK as an implant-abutment alternative to Ti6Al4V and Y-TZP [39]. Another study suggested that PEEK is the better clinical option when there is a need for maximizing the interaction between the soft tissue and implant components [40]. Porous PEEK was also found to outperform Ti-coated PEEK in osseointegration aspects [41].

### 2.2. Drawbacks of PEEK

PEEK, unfortunately, is also accompanied by several drawbacks when employed in dentistry. For instance, PEEK has been reported to be biologically inert due to its low surface energy challenging its clinical applications [36,42,43]. Poor osseointegration and inflammation reaction are the major complications found in implanted PEEK [25,36,44,45]. In dentistry, it is known that dental implants with insufficient bioactivity and osseointegration may develop severe implantitis, leading to the disintegration of implants. The in vitro studies conducted using human osteoblast [46] and human MG63 osteoblast-like [47,48,49,50] cells, and the in vivo studies conducted on dogs [51], rabbits [52], rats [53], and sheep [54], all, revealed lower bioactivity and osseointegration with unmodified PEEK compared to Ti [33].

In addition, PEEK is also inert to several chemical treatments (except for sulfuric acid) [55], rendering its resistant to surface modifications. This is undesirable because the surface modifications of PEEK are required to promote bioactivity and osseointegration. Furthermore, the low surface energy and chemical inertness of PEEK make bonding to other materials challenging. Specifically, in restorations, adherence of high-performance polymer PEEK to other materials, such as veneering composites, is desired (more in Section 3). Hence, surface modification strategies of PEEK have been of high research interest (more in Section 4).

## 3. Desired Properties of High-Performance Polymers in Dentistry

In addition to PEEK’s biocompatibility, superior mechanical, thermal, and chemical properties, it is desired of PEEK to have exceptional adhesiveness (i.e., bonding strength), bioactivity (e.g., osseointegration and antibacterial activity), and friction-wear resistant (i.e., resistance to tribocorrosion) when employed in dentistry

### 3.1. Adhesiveness-Bonding Strength

As mentioned in Section 2.2, polymeric materials in dentistry must have excellent bonding strength. In restorative dentistry, polymeric materials are expected to bond with other dental materials such as composite materials, resin cement, filler materials, veneering resin, etc. [2,56,57]. In prosthetic dentistry, bonding with both hard and soft tissues is highly desired [25]. Unfortunately, the low surface energy, the inertness, and the resistance to surface modification of PEEK lower the bonding ability of PEEK with other dental materials, bone, and soft tissue [58]. Many approaches are discussed in the literature to enhance the adhesive property of PEEK. These include adding polymeric adhesives, metal doping, polymeric resilient liners, polymeric conditioning compounds, and welding. Surface modification approaches have also been explored to enhance the adhesiveness of PEEK, which will be discussed in Section 4.

Adhesives function simultaneously as a bonding agent between composite resin and dental hard tissue (dentine, enamel), and as a shielding component to protect collagen fibers at the resin-dentine interface from deterioration through remineralization [59]. Adhesive materials, which include monomers, fillers, stabilizers, and initiators, must have adequate mechanical properties, durability, and a pleasant look [59,60]. In addition, the adhesive layer should prevent leakage in the case of shrinkage of restorative composites [2]. This adhesive binding layer integrity is crucial to determine the success of dental restoration.

The ability of an adhesive system to stand mechanical load during mastication is crucial in determining successful PEEK-supported implant prostheses. Good retention between PEEK and titanium bases requires strong adhesive and cohesive strength of the adhesive system. Yilmaz et al. [61] addressed how the adhesive system-cement combination impacts the retention force between titanium bases and PEEK specimens. Part of the credit for the successful bonding to PEEK goes to the ingredients and solvents in the adhesive solutions. Methylmethacrylate-containing adhesives have been reported to offer a long-lasting micromechanical interlocking and a potential chemical link between PEEK and resins [61].

Doping is another strategy to minimize bonding-related deterioration in restorative dentistry. Dentine demineralization caused by acid etching and increased resin infiltration promotes bonding between resin and dentine. Following the elimination of mineral ions during dentine demineralization, the collagen matrix becomes visible and exposed. The hybrid layer is then produced where the resin adhesive penetrates and polymerizes within the demineralized collagen. A fraction of demineralized and exposed collagen is prone to deterioration by host-derived matrix metalloproteinases and bacterial collagenases, which are present at the bottom of the hybrid layer, worsening the restorative bond. The bottom of the hybrid layer thus presents the weakest bond in the composite restoration [59]. For this, zinc (Zn) has been incorporated into polymer resin-based composites to significantly enhance their performance. It has been found that Zn incorporation strongly enhances the performance and durability of polymeric composites because of antibacterial and remineralizing activities, thus suppressing biofilm formation. Hence, Zn-doped polymeric resin-based composites provide minimized microleakage and long-lasting sealing [59].

**Figure 2:**
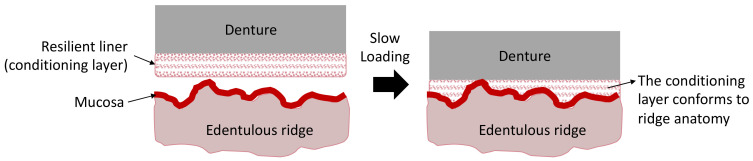
Schematic representation of the loading of the conditioning layer onto the ridge anatomy. The figure is redrawn with modifications from https://pocketdentistry.com/23-denture-liners/, accessed online on 14 November 2022.

Resilient liners are another example of soft and elastic adhesive agents, which have wide-range applications in the field of maxillofacial prostheses. They have been proven as an excellent clinical adjunct in patient care with persistent denture pain [62]. Plasticized acrylics and silicon elastomers are two polymer groups that have been used for long-term resilient liners. For example, poly(ethyl methacrylate), poly(butyl methacrylate), poly(methyl-hydrosiloxane), and divinylpoly(siloxane). Their usage may last for a few months and even years. While the viscoelasticity of the liner determines how well it can absorb masticatory stress, elastic behavior determines how well the liner expands the contact area beneath the denture base in response to the masticatory load, resulting in uniform pressure distribution. Resilient liners made of acrylic have higher elastic and viscoelastic behavior than those made of silicon. They have been reported to be more effective in relieving stress and improving patient comfort. However, they were found to be less durable than silicon-based materials [62]. There have been attempts to incorporate antibacterial agents such as nystatin and nanosilver into the liner materials to combat microbial colonization. However, it has been reported that these effects are transient [62]. In addition, considering dentures are frequently fabricated using CAD-CAM technologies [63], it is thus essential to study the adhering properties of resilient liners to polymer materials, such as PEEK. The use of resilient liner as a conditioning layer on the denture surface is illustrated in Figure 2.

**Figure 3:**
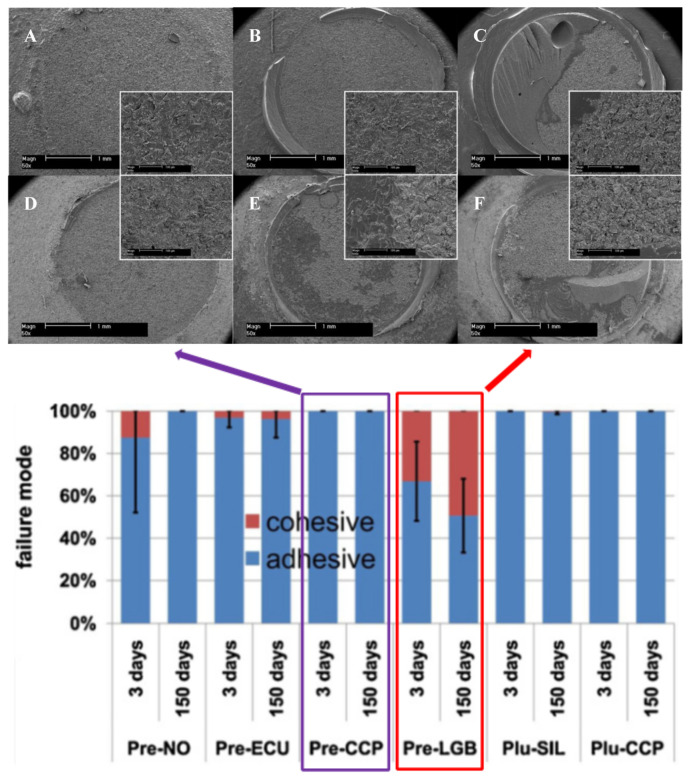
Above: SEM images of group samples measured after three days (**A**–**C**) and 150 days (**D**–**F**) with 50× and 500× magnifications. (**A**,**D**) depict spontaneously debonding, showing complete adhesive failure mode. (**B**,**E**) depict samples with medium tensile bond strength (TBS), showing residuals of primer. (**C**,**F**) depict samples with high TBS, showing residuals of primer and resin. In (**C**,**F**), a multifunctional resin varnish, including methacrylates as a conditioning layer, was applied to air-abraded PEEK surfaces. This conditioning layer demonstrated a long-lasting bond with the PEEK. Below: Failure modes of bonding groups. The figures were adopted and modified from the literature [65].

Surface conditioning is an approach where the surface is pre-treated with an adhesive layer to achieve durable bonding. Stawarczyk et al. explored the tensile bond strength between veneering compounds and PEEK at various pretreatment conditions and conditioning systems [64]. The pretreatments included air abrasion using different particle sizes (50 and 110 μm) and at various pressures (0.05, 0.28, and 0.35 MPa). The pretreatments were followed by conditioning with different commercial bonding liquids, and at the final step, the interface was veneered and aged. The results showed that the conditioning (adhesive system) significantly influenced the tensile bond strength, followed by air abrasion pressure. In contrast, the influence of air abrasion particle size was negligible. Similarly, Kern et al. suggested that conditioning PEEK surfaces with methylmethacrylate-containing primer after air abrasion significantly improved the tensile bonding strength of PEEK [65]. The authors show various adhesion failures between PEEK and a composite resin as presented in SEM images in Figure 3.

Rosentritt et al. studied shear bond strength between PEEK and veneering composites after surface roughening and conditioning. Enhanced bonding of the composites on PEEK surfaces was achieved when the surfaces were conditioned with either acetone- or phosphate-based methacrylate primers or were tribochemically treated. In addition, it was revealed that application of opaque materials increased the shear bond strength [66]. Escobar et al. [67] pre-treated PEEK surfaces with 98% sulfuric acid for 60 s (sulfonation process), followed by conditioning with a universal acidic adhesive for different conditioning durations, i.e., 0, 1, 3, and 5 min. The deposited adhesive was then polymerized under LED irradiation (1200 nW/cm^2^ for 60 s). The results showed that shear bond strength was increased as conditioning duration was increased, i.e., a ca. 4.95 MPa for 0 min to ca. 21.43 MPa for 5 mins, respectively.

**Figure 4:**
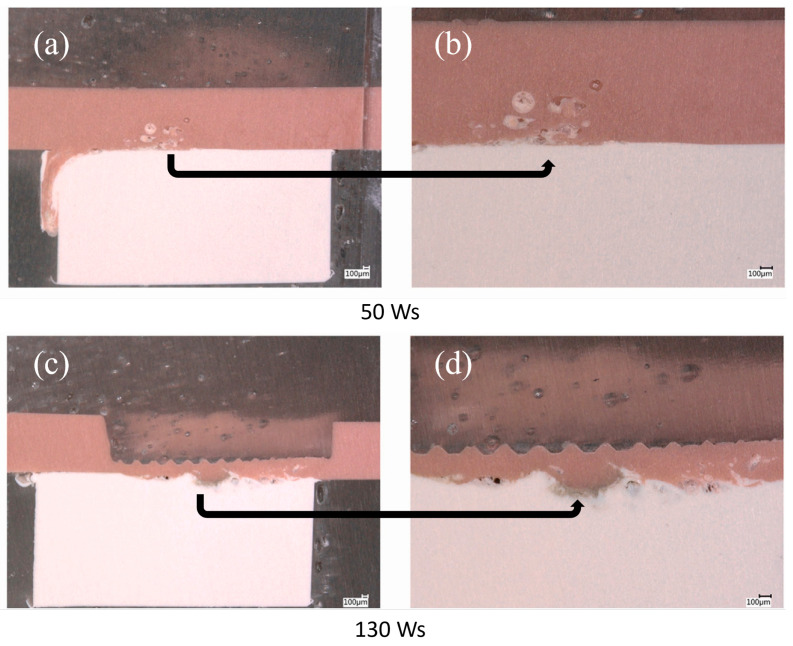
Representative cross sections of the welding joints of the red and white PEEK material combinations at 50 Ws (**a**,**b**) and 130 Ws (**c**,**d**). Figures (**b**,**d**) are the close-ups of (**a**,**c**), respectively. The figures were taken and modified from literature [68].

The bonding strength between polymeric dental devices is also influenced by their fabrication techniques. Ultrasonic welding is one of the practical methods employed to permanently join various dental PEEK pieces together in a denture structure [68]. The ultrasonic energy causes friction and hence heats the interface between the two interacting PEEK surfaces leading to the formation of a welder bond without melting the material. Abdulfattah et al. [68] found that when the welding energy was increased from 50 to 90 Ws, PEEK underwent higher surface deformation, leading to higher shear bond strength. However, an excessively high weld energy (130 Ws) significantly reduced shear bond strength due to a strong indentation and perforation brought on by sonotrode. Figure 4 shows the representative cross-sectional micrographs of welded red and white PEEK joints, using low (50 Ws) and high (130 Ws) weld energy. Thicker welding zone were observed at 130 Ws compared to 50 Ws, due to the higher sonotrode indentation depth. Along with the thicker weld zone, weak spots at the edges of the weld connections caused by the deep indentation were also seen.

### 3.2. Bioactivity (Osseointegration and Antibacterial Activity)

#### 3.2.1. Osseointegration

Osseointegration is when the implant directly interacts with the surrounding host bone until they are entirely stabilized. This process is necessary for a successful prosthesis [69]. Clinically, good osseointegration provides rigid and asymptomatic implant-supported prostheses during functional loading. Osseointegration in dentistry relies on the osseoconductivity of dental implant materials and bone regeneration [70]. Terheyden et al. [71] have visualized and disclosed the molecular and cellular communication during the osseointegration process after the installation of a dental implant. They observed four phases during osseointegration: hemostasis, inflammatory phase, proliferative phase, and re-modeling phase [72].

It is then clear that implanted dental devices should stimulate osseointegration. However, considering that PEEK is bioinert, osseointegration is challenging. Moreover, PEEK is not intrinsically osseoconductive [36]. Efforts to improve osseointegration of PEEK include the combination of surface modifications (it is discussed further in Section 4) and incorporation of bioactive materials, such as silicon nitrides [73], nano bio-glass [74], nanoporous lithium-doped magnesium silicates [75], mesoporous diopside [76], and biphasic bioceramics [77].

#### 3.2.2. Antibacterial Activity

Polymeric dental materials should have antibacterial properties because (1) the oral cavity is the house of a variety of microorganisms, including bacteria; (2) polymers which are rich in carbon and oxygen, tend to adsorb the microorganisms; and (3) the adsorbed microorganisms might lead to diseases such as periodontal inflammation and caries, e.g., *Candida* species are prone to adhere onto acrylic dentures [2,78]. Accumulating oral bacteria on dental material surfaces will lead to biofilm formation followed by bacterial infections. Oral bacteria can attach onto both hydrophobic and hydrophilic surfaces [78]. Further details related with biofilm formation on dental materials can be found in literature [78,79,80].

Strategies to incorporate antibacterial properties into polymeric dental materials include surface modifications and/or surface deposition of antibacterial agents. Surface modifications concentrate on designing surface topography (i.e., roughness), wettability (i.e., hydrophobicity, hydrophilicity), and surface free energy by means of physical, chemical, or mechanical methods [2,81]. Further details regarding surface modifications and how it influences the antibacterial performance of polymeric dental materials is discussed in Section 4.

Common antibacterial agents in dentistry include silver nanoparticles, antibiotics, quaternary ammonium salt, and bactericidal peptides [2,81]. Antibacterial agents display two killing mechanisms. First, the antibacterial active molecules are released from the surfaces into the surrounding medium over time. The second mechanism is where the antibacterial agents are bonded onto the surface, promoting bacterial remediation upon contact [82]. The drawback of the first system is the expenditure of antibacterial agents within the first week of implantation, resulting in depleted antibacterial activity over time [83,84]. Thus, the second mechanism (i.e., the contact-killing surfaces) is more beneficial and, when accompanied by a surface cleaning system, repels debris of dead bacteria from the surfaces [85].

In terms of PEEK in dentistry, silver is the commonly used antibacterial agent, which has antibacterial activity against Gram-positive and Gram-negative bacteria. Nano-silver ion is reported to have higher antibacterial activity than its counterpart silver ion due to its larger surface area and better ability to release the silver ion over a longer duration. Thus, surface coating of PEEK dental implants with silver nanoparticles can improve the success rate of the implantation and reduce the occurrence of peri-implantitis inflammation [86].

### 3.3. Tribology-Resistance to Tribocorrosion

Dentistry requires prosthetic devices to display biocompatibility, wear resistance, and withstand masticatory demands in corrosive environments [87]. Dental components are expected to damage when dental surfaces bump into or slide past each other. Although initially undetectable, the deterioration via wear could eventually cause severe mechanical failures [88]. Also, tooth wear occurs when the surfaces of dental hard tissue (dentine, enamel) and filling (restorative materials) rub up against one another. Ensuing tribological qualities have a significant impact on dental hard tissue durability. In practice, the tribological interface exists between dental hard tissue and dental instruments, implants, or prostheses that are in contact with the hard tissue [89]. Eventually, the evaluation of dental device performance depends on the simultaneous degradation (bio-tribo-corrosion) assessment of the materials [90].

A comprehensive tribological test to study the relationship between wear and friction of PEEK moving against steel at different length scales has been reported [88]. Different testing configurations with different nominal contact sizes were employed, i.e., block-on-ring, block-on-disc, cylinder-on-disc, cone-on-disc, block-on-flat, and single asperity micro scratching. The shear strength, calculated from single-asperity measurements, was divided with the effective yield pressure of the material to estimate the overall friction coefficient. In terms of wear performance, rotating contact and a smaller contact area resulted in a higher reduction in PEEK thickness under otherwise similar conditions, which can be attributed to wear and eventual removal of debris from the contact interface [88].

Further, PEEK appears to be a potential material for dentistry instruments such as oscillating tips for activating irrigants in endodontics, polymer burs in conservation, etc. The friction coefficients and specific wear rate of PEEK against human enamel/dentine were studied in various wet conditions [89]. It was observed that the average friction coefficient of wet dental hard tissue (enamel, dentine) against PEEK for a block-on-ring tribological test configuration was unaffected by the relative velocities within the experimental range (0.1–1 m/s). The stationary sliding friction coefficient varied between 0.54 and 0.58 for rotational velocities of 1 and 0.1 m/s, respectively. The specific wear rate of the dental hard tissue was substantially lower (0.9 mm^3^/Nm) than that of PEEK (80 mm^3^/Nm). Thus, PEEK appears to be a potential material for fabricating devices for selective caries excavation and for less invasive endodontic treatments.

Souza et al. [90] assessed the friction and wear behavior of PEEK matrix composites against alumina. The PEEK matrix composites containing natural amorphous silica fibers or particulate lithium zirconium silicate glass ceramics were employed. A reciprocating (alumina) ball-on-(PEEK matrix composites) plate tribometer was employed for wear testing at 37 °C in Fusayama’s artificial saliva. The sliding contacts comprising PEEK matrix composites reinforced with natural silica fibers showed the lowest friction coefficient (0.10) regardless of the fiber concentration. Compared to the unreinforced PEEK, the wear behavior of the PEEK composites was negatively impacted by the additional incorporation of lithium zirconium silicates.

Sampaio et al. [91] compared the abrasive wear resistance of PEEK and Ti6Al4V on three-body abrasion under various loads and hydrated silica content. To a cylindrical specimen of 8-mm diameter and 4-mm height, micro-scale abrasion tests were conducted using solutions containing various weight percentages of hydrated silica at 60 rpm and normal loads applied after 600 ball rotations. It was found that PEEK had lower wear resistance than Ti6Al4V, evident by a higher rate of volume loss for PEEK than Ti6Al4V.

During chewing and tooth brushing, exposed teeth or restorative surfaces in the oral cavity are prone to abrasive wear. Babaier et al. [92] investigated how the surface characteristics of PEEK-based CAD/CAM composite blocks were affected by food-simulating liquids to imitate the oral environment. PEEK-based specimens were exposed to water, 70% ethanol/water, and methyl ethyl ketone (MEK) at 37 °C for 1–7 days. No significant change in the surface roughness of the specimens (remained below 0.2 μm) was observed upon exposure to water and MEK. However, the surface roughness increased by a factor of four when the specimen was aged in 70% ethanol/water. This surface deformation led to the design consideration of covering every part of the denture framework with a veneer material for complete protection.

**Figure 5:**
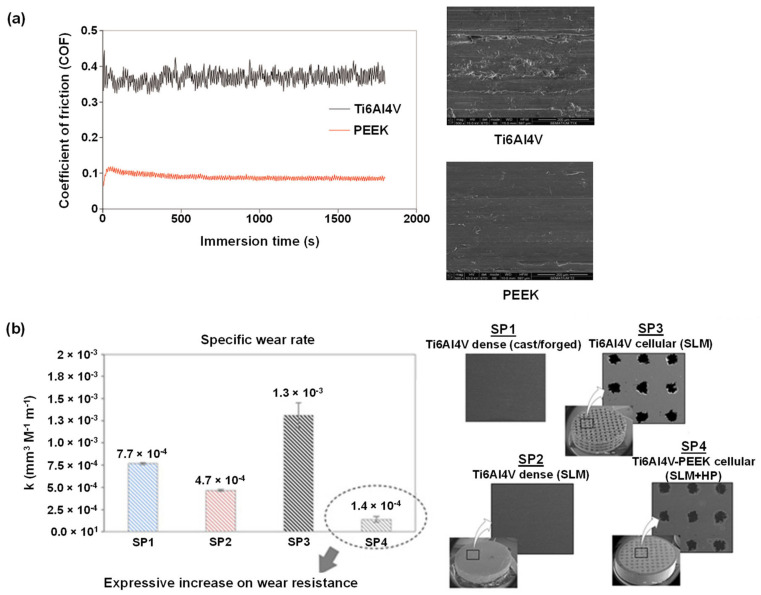
(**a**) Coefficient of friction as a function of immersion time of Ti6Al4V and PEEK (left) and the corresponding FEG-SEM images of the worn surfaces after reciprocating sliding tests against Al_2_O_3_ immersed in artificial saliva at 37 °C (right) [87]. (**b**) The specific wear rate of various Ti6Al4V-PEEK multi-material specimens against alumina normal load of 6 N at a frequency of 1 Hz and 3 mm of stroke length in Phosphate Buffer Solution at 37 °C (left) and the corresponding SEM images (right). SLM = selective laser melting, HP = hot pressing [96]. The figures were adopted and modified from literature [87,96].

Further, literature highlighted serious concerns about the toxic nature of the discharged wear particles into the human body [93,94,95]. For example, tissue inflammatory responses have been linked to wear debris and released ions from debris formed during bio-tribocorrosion of titanium-based prosthetic surfaces. On the other hand, PEEK is impervious to a wide range of both organic and inorganic fluids; thus, PEEK can shield metal surfaces from corrosion when used as a veneering material. Sampaio et al. [87] examined the tribocorrosion behavior of a hybrid structure composed of veneering PEEK to Ti6Al4V in modified Fusayama’s artificial saliva. Tribocorrosion studies report PEEK demonstrates a reduced coefficient of friction against Al_2_O_3_ than Ti6Al4V, i.e., 0.07 vs. 0.36, respectively (Figure 5a). Thus, the PEEK veneer can be employed to prevent corrosion and wear of Ti6Al4V.

In contrast to the above-described study [87], where PEEK was employed as a veneer, Bartolomeu et al. [96] fabricated a multi-material PEEK-Ti6Al4V structure utilizing selective laser melting and hot pressing techniques (Figure 5b). The dynamic coefficient of friction under reciprocating sliding was evaluated, and open-circuit potential was measured before, during, and after sliding the contact. Compared to mono-material Ti6Al4V, the multi-material PEEK-Ti6Al4V specimens showed improved wear resistance and a lesser tendency to erode.

It can be concluded that, in general, the tribological qualities of PEEK are better than titanium, and experimental research that showed otherwise does exist [91]. Further, considering the toxicity from titanium wear particles (as evidenced by volume loss), PEEK presents a preferable option.

## 4. PEEK Surface Modifications

The surface design of dental materials dictates the dental materials' performance in relation to their bonding strength, osseointegration, antibacterial properties, and tribological properties. We note that in this review, we will especially highlight the effects of surface treatments on the first three properties. Further, this review section will focus on two surface modification approaches, i.e., surface topography and surface chemistry, where the combination of these two properties influence surface wettability. Different opinions regarding which surface property is more critical do exist. Cruz et al. stated that surface chemistry plays a more important role than surface topography [38]. On the other hand, Torstrick et al. suggested that surface structure and feature are more important than implant surface chemical composition [74]. Nevertheless, surface modification treatments that alter these surface properties can be classified into two groups, i.e., chemical and physical treatments. Such treated surfaces can then be further modified via biocompatible agents, for example, by depositing bioactive compounds such as growth factors, osteogenic agents, and antibacterial agents [81]. Modifications also involve (nano)composite formation, material blending, and other techniques, however, they are not the focus of this study, as these approaches modify the bulk properties of PEEK. This review aims to discuss surface modification techniques solely.

### 4.1. The Importance of Surface Wettability

Cellular attachment in osseointegration requires a moderate hydrophilic surface [36]. Lang et al. [97] showed that the degree of osseointegration on hydrophilic surfaces was superior to that of hydrophobic surfaces (observation was conducted within 2–4 weeks in human clinical studies). Sartoretto et al. [98] also observed similar phenomena in animal studies. The preference for a hydrophilic surface for successful osseointegration in dental applications can be summarized as: (1) a hydrophilic surface is preferable for protein adsorption, and even more, the adsorbed proteins can maintain their conformation and function, and (2) prior protein adsorption encourages cell attachment and migration onto the implant surface as well as osteoblast differentiation and maturation [99]. Animal studies by Shwarz et al. [100] revealed that hydrophilicity promotes early osseointegration that can be identified by observing an increase in osteocalcin, bone density, and bone-to-implant-contact values. PEEK, however, is hydrophobic due to its low surface energy [36,42,43]; hence, surface modifications are necessary to render hydrophilicity for PEEK surfaces.

Surface wettability also influences bacterial adhesion, where the attachment depends on the species of the bacteria, i.e., hydrophobic bacteria prefer hydrophobic surfaces, while hydrophilic bacteria prefer hydrophilic surfaces [101]. While more than 500 bacteria species circulate in the oral cavity, the main contributor to chronic periodontitis is *Porphyromonas gingivalis* [102]. *P. gingivalis* has hydrophobic properties, and thus it’s attachment to the hydrophilic surface is discouraged [103]. Hence it is expected that surface modification to enhance surface hydrophilicity will be beneficial for promoting osseointegration and repressing *P. gingivalis* (and other hydrophobic oral bacteria) adhesion. Nevertheless, it is important to note that the interaction between bacteria and surface is a rather complex phenomenon and is influenced by several factors, including external shear stress, other microorganisms, salivary pellicle, surface topography, etc. [104]. In addition, whether hydrophilic implants are long-term beneficial, is a question that researchers must investigate.

### 4.2. The importance of Surface Topography

Surface roughness can induce a better bonding strength between polymeric surfaces and other dental materials. Many researchers have explored the relationship between adhesiveness and surface roughness. For example, bonding between veneering materials and PEEK surface has been improved (as evidenced by an increase of shear bond strength) by both mechanical and chemical approaches [6], such as airborne-particle abrasion [105,106,107,108,109,110,111], silica coating [105,106,108,110], laser etching [105,106], plasma treatment [107,111,112], organic solvent (acetone) treatment [105], and acid (H_2_SO_4_, piranha) etching [105,108,109,110]. Shabib et al. [113] examined the effect of different PEEK surface modifications on the attachment strength of composite resin. The PEEK surfaces were treated with photodynamic therapy, Neodymium-doped yttrium orthovanadate laser, sulphuric acid, and sandblasting. The laser treatment generated the highest surface roughness (R_a_ = 15 μm) and, subsequently, the strongest shear bonding (16 MPa). The shear bond strength was further enhanced by adding a resin cement as an adhesive between the PEEK surface and the composite resin.

Aside from bonding strength, surface topography also determines cellular behavior and activity, such as cell adherence and spreading [114]. In vivo studies by Hieda et al. and Yuan et al. revealed that porous surfaces encourage new bone ingrowth leading to enhanced implant stability. On the contrary, only an insignificant amount of new bone ingrowth was observed on smooth and denser surfaces [115,116]. Evan et al. added that less fibrous encapsulation was observed on porous surfaces, which further improved implant stability [117]. This implant stability is important not only during osseointegration but beyond. The pore size is shown to be a critical parameter as it relates to cell penetration and vascularization, which is beneficial for dental material integration with surrounding tissue [118,119]. In addition to pore size, pore permeability also contributes to determining cell behavior, vascularization, and nutrient delivery [120]. However, the incorporation of surface porosity in PEEK can impair mechanical properties, such as decreased elastic modulus and yield strength [121]. This problem has been approached by the addition of additive fillers (in order to increase the mechanical properties of porous PEEK) [121,122]; however, the results are still far from optimum. Optimizing the structural design of PEEK-based dental material and its manufacturing methods may be an option to solve this drawback [123].

In regard to surface roughness, some studies suggested that nanoscale surface roughness may not be sufficient to promote strong osseointegration compared to micron-scale roughness [81,124]. Another study showed that nanoscale roughness could alter surface free energy, thus enhancing cell growth and osteoblastic differentiation [125]. In comparison, microscale roughness increased the fibrin matrix formation, which is essential for osteogenic cells and bone matrix deposition [81].

One technology that can be used to directly fabricate PEEK-based dental materials with various surface roughness and intrinsic porous structure is via 3D printing. Most common 3D printing methods used for PEEK manufacturing are selective laser sintering (SLS) and fused deposition modelling (FDM) [126]. Few studies show improved osseointegration on 3D-printed PEEK surfaces when incorporated with desired surface roughness and distinct patterns (peaks and valleys) [127,128]. Other studies showed a preference for cells to settle at the surface grooves, probably because such sites promoted cell-cell contact, enhancing cell viability [129,130].

However, surface roughness has also been shown to assist the buildup and adherence of bacterial plaque on dental materials. The clinically acceptable surface roughness for dental prostheses is R_a_ = 0.2 μm, where no significant biofilm formation was observed below this critical value. However, the biofilm formation is shown to accelerate when the surface roughness exceeds the R_a_ threshold (0.2 μm) [29,131]. The surface roughness provides protection from external shear forces and presents a larger surface area to promote bacterial attachment [101]. Nevertheless, the biofilm formation does not strictly follow this rule, considering the complexity of the bacterial attachment process that is influenced by environmental conditions and the dimensions of bacteria strains (size and shape) relative to surface nano-microstructures [132]. When low surface roughness is required, polishing can be used instead of roughening [29]. According to several reports, the final surface roughness and surface quality rely on polisher type, velocity, contact pressure, polishing media (paste, dry, or wet), and polishing protocols (chairside or laboratory) [29]. According to a published report [133], chairside polishing resulted in lower surface roughness values compared to the laboratory polishing procedure.

Thus, designing dental device surfaces with optimum surface topography that promotes bonding strength and osseointegration while decreasing bacterial attachment is challenging. 

### 4.3. Chemical Surface Modifications of PEEK

**Figure 6:**
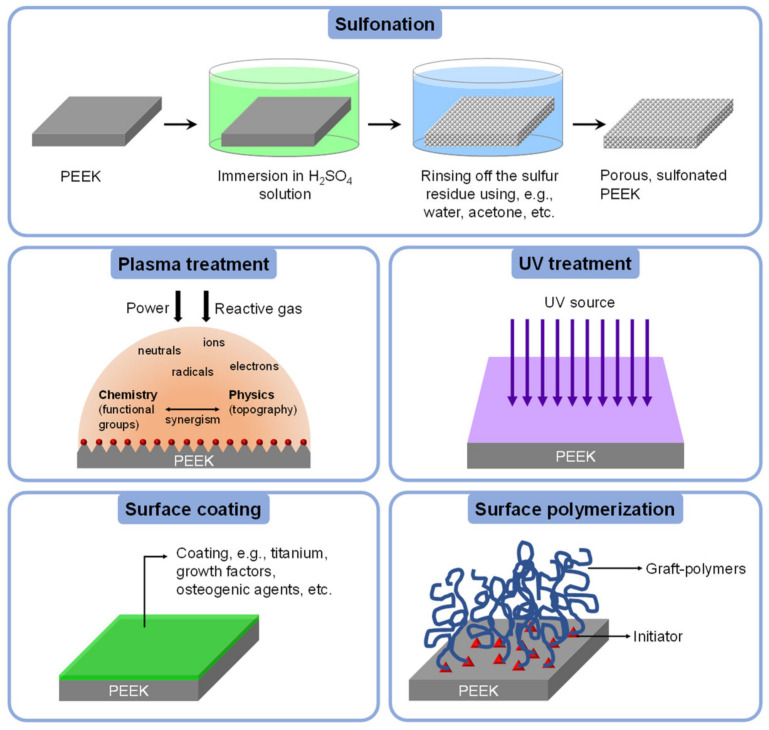
Illustration of chemical surface modifications of PEEK. The illustration is inspired by previously published literature [134,135,136].

In this section, chemical techniques used for PEEK surface modification are categorized into two groups, i.e., the sulfonation process involving concentrated strong acid and non-sulfonation methods. The scope of the corresponding techniques is illustrated in Figure 6.

#### 4.3.1. Sulfonation

One of the most reported methods to modify PEEK surface is sulfonation by chemical treatment, followed by antibacterial and/or osteogenic agents deposition [114,137,138,139,140]. Sulfonation is conducted in concentrated sulfuric acid (H_2_SO_4_), resulting in a sulfonated PEEK surface with a 3D porous structure of nano- to micro-scale roughness and generates -SO_3_H functional groups. The excess sulfur is rinsed out in the post-treatment methods, such as hydrothermal treatment [141], acetone rinsing [142], and NaOH rinsing [143]. Ma et al. have investigated the effect of sulfonation reaction time (0.5, 1, 3, 5, and 7 min) and post-treatment methods on the generated surface morphology and cell attachment behavior on PEEK. The results showed that reaction time of 5 min was optimum, and the post-treatment methods were equally effective in removing the excess sulfur, and cell reaction was not affected by the different post-treatment methods [144]. Zhao et al. [145] reported that porous sulfonated PEEK consecutively washed with water and acetone showed improved cytocompatibility compared to water-only wash. It was speculated that the latter case resulted from residual sulfur on the PEEK surface. The cytocompatibility was evaluated in terms of osseointegration and bone-implant bonding strength in vivo, as well as induction of pre-osteoblast functions such as initial cell adhesion, proliferation, and osteogenic differentiation in vitro.

The drawback of the sulfonation process is the undesired decrease of surface hydrophilicity [25]. However, some researchers have proven otherwise. Wang et al. decreased PEEK water contact angle from 78° to 37° by immersing the PEEK surface in NaOH after 20 s of sulfonation process [143]. Cheng et al. enhanced the wettability of sulfonated PEEK by immersion in NaOH for 24 h. This extensive NaOH treatment did not change the surface morphology nor the surface chemistry, as shown from SEM and water contact angle measurements, respectively [141]. Miyazaki et al. improved the hydrophilicity of sulfonated PEEK substrates by 24 h immersion in 1 mol/L CaCl_2_ solution [146].

Sulfonation process can be combined with other surface modification methods such as plasma treatment, UV treatment, and additional material deposition (e.g., polymer layer and bioactive compounds such as antibacterial agents and growth factor) [25,147,148]. It was reported that cell response on the PEEK surface could be improved by combining sulfonation with the plasma treatment process, increasing surface hydrophilicity [147,148]. Sulfonated PEEK dental implants have been functionalized with boron-doped hydroxyapatite nanoparticles. Compared to unmodified PEEK, sulfonated PEEK substrates showed a significant improvement in adhesion, proliferation, and osteogenic differentiation of periodontal ligament cells in in vitro cell culture experiments [34].

**Figure 7:**
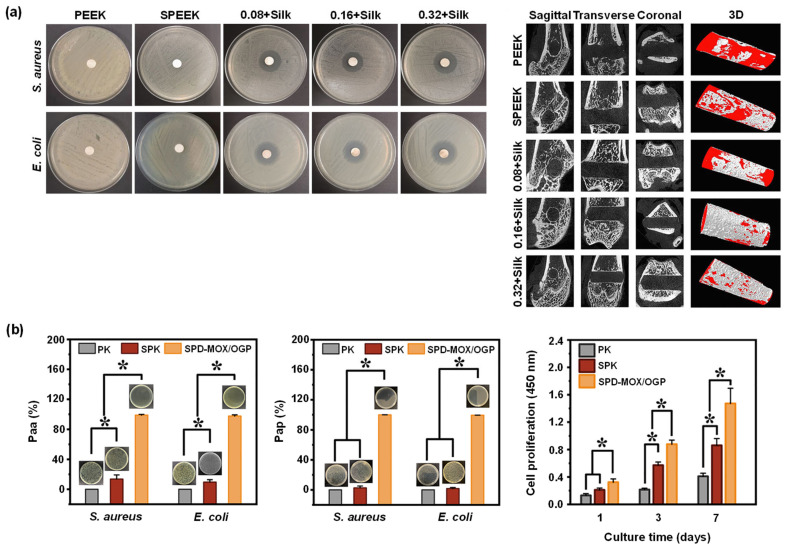
Sulfonation treatment of PEEK surface. (**a**) Sang et al. compared the biological properties of untreated PEEK (PEEK), sulfonated PEEK (SPEEK), and sulfonated PEEK coated with SrCO_3_-poly(dopamine) + gentamicin-silk protein (*n* + silk, where *n* defines the concentration of SrCO_3_-poly(dopamine), i.e., 0.08 mg/mL, 0.16 mg/mL, and 0.32 mg/mL, respectively). Left: in vitro antibacterial zone inhibition tests. Right: in vivo osteogenesis as imaged using µCT [138]. (**b**) Biological properties of untreated PEEK (PK), mildly sulfonated PEEK (SPK), and SPK layered with poly(dopamine) and loaded with moxifloxacin hydrochloride and growth peptide (SPD-MOX/OGP). Paa = antibacterial percentage of adherent bacteria. Pap = antibacterial percentage of planktonic bacteria. * *p* < 0.05 [140]. All figures were re-printed with permission from the literature [138,140].

Sulfonated PEEK has also been embedded with strontium carbonate SrCO_3_ nanoparticles, a bone formation promotor [149]. To allow a steady release of strontium ions, poly(dopamine) was utilized as an intermediate to embed the SrCO_3_ nanoparticles in the microporous sulfonated PEEK surfaces. The surfaces were then further coated with gentamicin-silk protein, where the release of gentamicin will induce antibacterial properties. Silk proteins were employed as they present abundant functional groups to bind to drug (such as doxorubicin and gentamicin) carriers [150]. In vitro experiments were carried out using bacteria *Staphylococcus aureus* and *Escherichia coli*, and cells RAW246.7 and hBMSCs, while in vivo tests were conducted on rats. Both in vitro and in vivo studies demonstrated that the modified PEEK material exhibit robust antibacterial and osteogenic properties (Figure 7a left and right, respectively) [138].

Gao et al. [140] constructed a relatively uniform porous structure on PEEK surfaces using mild sulfonation method, i.e., by ultrasonication of the PEEK substrates in concentrated H_2_SO_4_ at 25 °C for 30 s, followed by ultrasonication in de-ionized water, acetone, and ethanol for 30 min each. The samples were finally hydrothermally treated at 100 °C for 4 hours to eliminate any remaining sulfur compounds. The resulting porous sulfonated PEEK samples were coated with a poly(dopamine) layer, which serves as a diffusion barrier modulating the release rate of bioactive drugs through the pores. Moxifloxacin hydrochloride and growth peptide were then co-loaded, acting as antibacterial and cell-attractive agents. The in vitro studies showed dual-functional PEEK surfaces demonstrated an exceptional (1) antibacterial effect against adherent and planktonic bacteria (Figure 7b left and middle, respectively) and (2) improvement in the MC3T3-E1 cell adhesion, proliferation, and osteogenic differentiation (Figure 7b right). In vivo studies in rats showed enhanced osseointegration, and bacteriostatic properties.

Luo et al. [114] blended the bioinert PEEK with tantalum (Ta; an osteogenic agent) nanoparticles to prepare Ta/PEEK-based composites. The composites were then exposed to concentrated H_2_SO_4_ (sulfonation process) and loaded with an osteogenic and antibacterial isoflavone, genistein. Characterizations of the composites showed that the surface of Ta/PEEK was smoother, while the surface of sulfonated Ta/PEEK was rough with micropores (size was ca. 2 μm) holding Ta nanoparticles (size was ca. 50 nm). Furthermore, it was observed that loading genistein into the micropores reduced the surface roughness. In vitro bacterial assay was performed using *S. aureus* and *E. coli*, while cell culture assay was performed using MG63 cells. In vivo test was conducted on rabbits. The results revealed that the genistein-loaded microporous Ta/PEEK composites exhibited dual-functional properties, i.e., osteogenic and antibacterial interface, and promoted bone regeneration and osseointegration, indicating its tremendous potential for bone substitution.

Liu et al. [139] fabricated a macroporous PEEK scaffold using 3D printer, sulfonation, and UV-induced graft polymerization. The resulting scaffold was then functionalized with methacrylated chitosan/polyhedral oligomeric silsesquioxane nanocomposites. For the growth of new bone, 3D printing offers a stereoscopic framework with a macroporous structure, methacrylated chitosan provides a bioactive microporous surface for cell adhesion and spreading. The polyhedral oligomeric silsesquioxane nanoparticles serve as fillers that physically and chemically strengthen the hydrogel network and stimulate calcium deposition and osteogenic differentiation in cells. In vitro experiments using rat bone marrow mesenchymal stem cells (rBMSCs) showed that the 3D porous structure and the modified PEEK scaffold's bioactive surface not only offered favorable conditions for cell adhesion and proliferation but also boosted osteogenic differentiation of the rBMSCs. In addition, in vivo studies in rats showed considerable stimulation of bone regeneration.

Ekambaram et al. [151] strived to increase the surface area per volume ratio of guided tissue regeneration membrane for periodontal treatments by electrospinning of sulfonated PEEK that has been blended with aminated zirconia nanoparticles and curcumin. The electrospinning process was performed in dimethylformamide solvent. Antibacterial test and in vitro biocompatibility assay were carried out against *Streptococcus oralis*-2696 and Vero cells, respectively. The PEEK-based nanofibrous substrates with incorporated aminated zirconia was found to have potential for achieving strong cell attachment and electrically stimulating cells. The sustained release of curcumin from the substrates increased cell viability, antibacterial capability, cell proliferation, and the critical requirement for a periodontitis healing process.

The effect of sulfuric acid concentration on the adhesiveness of PEEK with resin composite was recently investigated. Five concentrations of sulfuric acid, i.e., 70%, 80%, 85%, 90%, and 98%, were employed to treat the surfaces (for 60 s). The measured bond strength values ranged from 1.37 MPa, 17.47 MPa, 21.53 MPa, 26.68 MPa, and 27.36 MPa, respectively (untreated PEEK bonding strength was 1.75 MPa). Hence, concentrations of sulfuric acid at 90% and 98% are optimum for higher bonding strength [152]. These results contrast Escobar et al. measurements, which showed the PEEK treatment with 98% sulfuric acid for 60 s led to a minimal increase in shear bond strength, i.e., 4.95 MPa [67]. This might result from different PEEK surface pretreatment (i.e., polishing conditions), post-treatment conditions, and/or other process parameters. Nevertheless, further research is necessary to establish the optimal acid concentration for enhancing bonding strength of PEEK. The effect of the duration of sulfonation on bonding strength between PEEK and veneering resin has also been investigated. Two groups of PEEK, 3D-printed and milled substrates were exposed to sulfonation for 0, 5, 30, 60, 90, 120, and 300 s. The highest shear bond strength for 3D-printed PEEK was achieved after 30 s of acid treatment (ca. 27.9 MPa), similar to milled PEEK (also after 30 s treatment). The bonding strength of milled-PEEK sulfonated for 5 to 120 s did not show a significant difference (all substrates had bonding strength above ca. 29 MPa). Thus, it was concluded that the optimal sulfonation duration was 30 s for 3D-printed PEEK and 5–120 s for milled PEEK, while a longer sulfonation duration led to a slightly decreased bonding strength [153].

#### 4.3.2. Non-Sulfonation

Despite the numerous success stories of sulfonated PEEK in vitro and in vivo, when combined with other surface modification techniques and with drugs and/or bioactive agents coating [114,137,138,139,140], sulfur residues can be a considerable drawback. Brum et al. [137] compared the surface of sulfonated PEEK with unmodified PEEK, which showed (1) lower wetting, (2) lower cell proliferation, and (3) lower metabolic activity of fibroblasts after 1 and 3 days of incubation. The poor cytocompatibility of sulfonated PEEK materials was most likely caused by the leakage of sulfonic chemicals into the surrounding medium. Although sulfonation procedures can be optimized to reduce the risk of sulfur residue and increase the biocompatibility of sulfonated PEEK, many researchers have offered methods to modify PEEK surfaces without sulfonation [154,155,156,157].

##### Plasma Treatment

The fourth state of matter, plasma, can break covalent bonds of the material surface. This will lead to surface topography, chemistry, and wettability changes and disrupt the polymer chains at the interface [156,158]. The impact of plasma on surface properties depends on the gas type and plasma conditions. Oxygen plasma treatment introduces carboxyl and hydroxyl functional groups; hydrogen plasma treatment leads to single-bond hydroxyl groups, while ammonia and nitrogen plasma treatment introduces nitrogen-containing functional groups at the surface. Further, water plasma treatment generates hydroxyl functional groups [42,158,159]. Thus, it is expected that the effect of different plasma treatments on PEEK’s optimum surface roughness (hence the bioactivity of the modified surfaces), shows inconsistent data [25].

**Figure 8:**
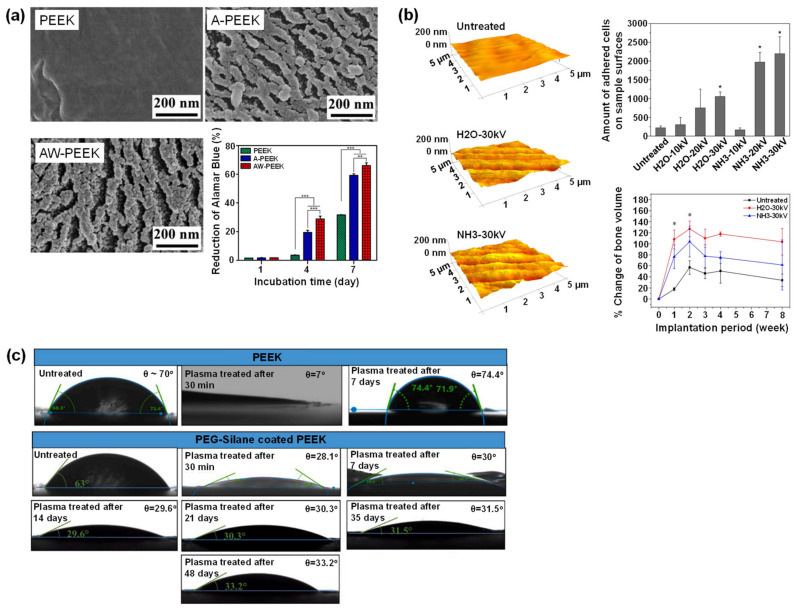
Plasma treatment of PEEK surface. (**a**) The work from Wang et al. showedsurface morphologies (characterized using field emission scanning electron microscopy) of untreated PEEK (PEEK), PEEK treated with argon plasma (A-PEEK), and PEEK treated with a mixture of water vapor and argon plasma (AW-PEEK). The Alamar Blue Assay was quantified against MC3T3-E1 cells (** *p* < 0.01, *** *p* < 0.001). A higher value of Alamar Blue Reduction indicates higher viability. (**b**) Zhao et al. showed AFM images and quantitative bioactivity analyses (MC3T3-E1 pre-osteoblast cell adhesion (* *p* < 0.05) and % change of bone volume of rats (* significantly higher bone formation after 1–2 weeks implantation)) of untreated PEEK, PEEK treated with H_2_O plasma and PEEK treated with NH_3_ plasma. (**c**) Sundriyal et al. compared the time-dependent water contact angle for plasma-treated PEEK and plasma/PEG silane-treated PEEK. All figures were reprinted with permission from the literature [156,158,166].

Fu et al. used different gas types of low-pressure plasma treatments and observed negligible surface roughness change. However, surface hydrophilicity and cell adhesion significantly improved when hydrogen was mixed with oxygen [159]. On the contrary, Wang et al. showed that osteoblast growth response and early osteogenic differentiation of MC3T3-E1 and rBMSCs were enhanced by plasma immersion ion implantation treatment using a gaseous mixture of water vapor and argon (Figure 8a). The plasma treatment introduced hydroxyl groups and a ravined nanostructure with an arithmetic average roughness of 15.3 nm [158]. Similarly, Zhao et al. [156] used plasma immersion ion implanter in H_2_O or NH_3_ plasma to modify PEEK surfaces. In vitro and in vivo tests were performed using mouse MC3T3-E1 pre-osteoblast and rats, respectively. The results showed that biological interactions on the modified PEEK surfaces, such as cell adhesion, proliferation, osteogenic differentiation, and new bone formation in vivo, were significantly improved due to the introduction of nitrogen- and/or oxygen-containing functional groups. And the enhanced surface hydrophilicity and surface roughness (R_a_ < 15.7 nm was found to be effective in promoting osseointegration). Higher voltages in the studied plasma voltage range (10–30 kV) resulted in better cell-PEEK interaction (Figure 8b). Furthermore, the surface plasma treatment approach did not alter the inherent mechanical properties of PEEK.

Waser-Althaus et al. [160] also found better cell adhesion, cell proliferation, and osteogenic differentiation due to the increased surface hydrophilicity for oxygen or ammonia plasma-treated PEEK surfaces. The treatment generated pillar-like nanostructures, whose dimensions depend on the gas type and the plasma power. The nanostructures with sizes in the range of 10 nm showed enhanced bioactivity compared to surfaces with higher roughness [160]. Contradictive results by Gan et al. showed an increase in surface roughness led to better cell response. PEEK surfaces exposed to nitrogen plasma immersion ion implantation resulted in nanostructured PEEK with roughness R_a_ = 436, 443, and 608 nm. Higher surface roughness showed remarkable improvement in cell proliferation, cell viability, and alkaline phosphatase (ALP) activity [161].

The impact of plasma treatment on the adhesion properties of PEEK has been positively proven [162]. It was suggested that exposing PEEK surfaces to oxygen plasma (low pressure) for 35 min could significantly improve the shear bond strength between PEEK and veneering composites [107]. Argon-plasma-treated PEEK also showed excellent enhancement of shear bond strength to dental cement [58]. Recently, Younis et al. investigated the impact of various type of gas plasma on the shear bond strength between veneering resin and PEEK. The results revealed that nitrogen plasma treatment gave the highest shear bond strength (ca. 10.04 MPa), followed by argon (ca. 9.56 MPa), air (ca. 9.27 MPa), and oxygen (ca. 8.59 MPa). The shear bond strength of untreated group itself was ca 5.38 MPa. Thus, surface plasma treatment can also be employed to increase the bond strength between PEEK and veneering resin [163].

Plasma treatment is usually combined with other surface modification methods for PEEK. For example, Przykaza et al. exposed PEEK surfaces to a low-temperature air plasma (20 °C, 0.2 mbar) followed by chitosan coating (100–300 kDa with 82% deacetylation degree) layered by a ternary Langmuir-Blodgett (lipid-sterol, peptide) film containing cyclosporine A, 1,2-dipalmitoyl-*sn*-glycero-3-phosphocholine, and cholesterol. This multilayer hybrid system showed accelerated osseointegration and drug (cyclosporine A) delivery properties [164]. Further, plasma treatment combined with hydrofluoric acid treatment showed improved cell adhesion, spreading, proliferation, and ALP activity of the resulted fluorinated PEEK. In addition, the fluorinated PEEK exhibited antibacterial activity against *P. gingivalis* [165].

Sundriyal et al. [166] combined oxygen plasma and PEG coating for PEEK surface treatment. The water contact angle of plasma-PEG-treated PEEK reduced from 70° to 28.1° after 30 min and maintained at 33.2° after 48 days. In contrast, plasma-treated PEEK without subsequent PEG coating failed to retain the surface hydrophilicity. It was reported that immediately after the plasma treatment, the water contact angle reached 7° and recovered to 74.4 ° only after 7 days (Figure 8c). Combined plasma-PEG-treated PEEK also showed durability of average roughness. This treatment increased the average roughness from 12.42 to 16.66 µm and slightly changed to 16.06 µm after 48 days.

##### UV Treatment

UV treatment of PEEK material aids surface hydrophilicity enhancement [167,168]. In vitro studies on UV-treated PEEK surfaces using fibroblasts from mouse and human gingival showed that UV treatment benefits early attachment and proliferation of soft tissue cells [169]. Another in vitro experiment using human dental pulp stem cells on UV-treated PEEK surfaces improved cell attachment and osteogenic differentiation [170]. Furthermore, UV irradiation can also be used to facilitate polymer grafting on PEEK surfaces using surface photopolymerization. This will be discussed further in Section Surface Polymerization.

##### Surface Coating

PEEK surfaces have been coated with various materials to improve it’s performance in dentistry [171]. Oladapo et al. [172] fabricated microporous PEEK using 3D printing and covered the PEEK surface with calcium hydroxyapatite to accelerate osseointegration. In addition, this technique enhanced the mechanical strength of the modified PEEK. Coating with hydroxyapatite can also improve the biomechanical properties of PEEK-based implants. PEEK coated with nanocrystalline hydroxyapatite had a significant increase of biocompatibility and removal torque [173].

Wei et al. manufactured porous PEEK scaffolds with a pore size of 400 μm and a porosity of 50% using the 3D printing. The porous surfaces were then functionalized with poly(dopamine) coating chelated with Mg^2+^ ions. The surface coating was applied by immersing the porous PEEK scaffolds in a solution (pH = 8.5) of Tris (hydroxymethyl)aminomethane and dopamine, followed by another immersion in a solution (pH = 8.5) containing Tris (hydroxymethyl)aminomethane and MgCl_2_. This approach significantly increased the hydrophilicity of the PEEK. In vitro studies using MC3T3-E1 cells and HUVEC lines showed that the bioactive coatings containing Mg^2+^ on porous PEEK scaffolds increased cell proliferation, cell adhesion, osteoblast differentiation, mineralization, and vascularization. In vivo studies were carried out by implanting the modified PEEK in rabbits. The results demonstrated that the release of Mg^2+^ promoted bone ingrowth inside the porous PEEK scaffolds by promoting early vascular ingrowth. In addition, the poly(dopamine) coating improved the poor interfacial osseointegration of the PEEK [155].

Another approach for PEEK surface coating is electron beam deposition. Pure titanium coating deposited on PEEK surface using this method significantly improved the surface wettability of PEEK. Cell (MC3T3-E1) proliferation and cell differentiation (measured using alkaline phosphatase assay) on Ti-coated PEEK doubled compared to uncoated PEEK. Animal studies demonstrated that osseointegration level on Ti-coated PEEK implants was higher than that on uncoated PEEK implants [174]. Similarly, nanoporous Ti-coated PEEK promoted immobilization of bone morphogenetic protein-2 (BMP-2; growth factor involved in the differentiation of host progenitor cells to induce bone formation [175], improving the biocompatibility and osseointegration of PEEK implants. The nanopores were created by anodizing the Ti coating, which had been deposited on the PEEK surface using electron beam deposition technique. The biocompatibility was measured in vitro through cell attachment, proliferation, and differentiation. The osseoconductivity, when measured in vivo, doubled compared to uncoated PEEK [176].

##### Surface Polymerization

Surface polymerization facilitates surface modifications, especially in terms of surface chemistry. The classical route to conduct surface polymerization is atom transfer radical polymerization (ATRP) and photopolymerization, the details of which will be discussed below.

*Surface atom transfer radical polymerization (ATRP)*

Surface ATRP allows the formation of polymer brushes with controlled topologies and molecular weight [177]. However, only a handful of research was conducted in this field [178]. Yameen et al. grafted three different monomers, i.e., potassium 3-(methacryloyloxy)propane-1-sulfonate (MPS), monomethoxy-terminated oligo(ethylene glycol)methacrylate (MeOEGMA), and N-isopropylacrylamide (NIPAAm) onto activated PEEK surfaces. The PEEK surface activation was achieved via a two-step process: (1) reduction of keto groups to hydroxyl groups, and (2) attachment (covalently) of 2-bromoisobutyryl groups as ATRP initiator. PEEK modified with polyMPS brushes showed electrostatic interaction with rhodamine 6G, surfaces grafted with polyMeOEGMA brushes were *E. coli* repellent, while PEEK with polyNIPAAm brushes was thermally responsive with switching between hydrophobicity and hydrophilicity [179]. The polyMeOEGMA-modified PEEK surface is interesting for dental applications among the three modified surfaces. A similar approach, i.e., grafting poly(ethylene glycol)methacrylate onto the PEEK surface, improved surface wettability [180].

*Surface photopolymerization*

**Figure 9:**
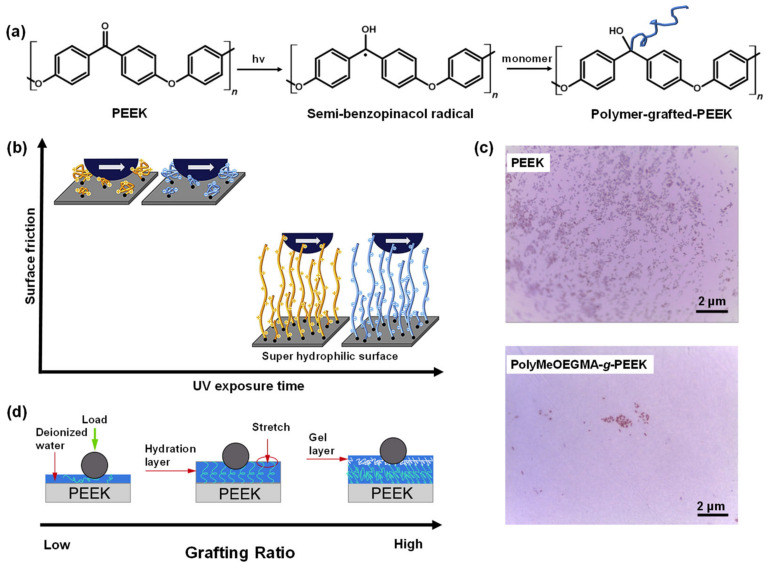
Surface photopolymerization of PEEK. (**a**) Mechanism of PEEK surface modification with grafted polymer chains via self-induced photopolymerization. (**b**) The effect of UV exposure time on surface friction of PEEK grafted with electrolyte polymer brushes. (**c**) Light microscope images of untreated PEEK and PEEK grafted with PolyMeOEGMA showed the antifouling characteristics of PolyMeOEGMA-*g*-PEEK against *E. coli*. (**d**) Illustration of tribological properties of PAA-*g*-PEEK with different grafting ratios under water lubrication. (**b**–**d**) were reprinted with permission from the literature [183,186,187].

UV- or photo-initiated polymerization of PEEK is made possible by the presence of diphenylketone groups (act as photoinitiators) at the polymer backbone chains. Upon exposure to photo-irradiation, semi-benzopinacol radicals are formed at the PEEK interface (Figure 9a) [181]. Kyomoto et al. grafted 2-methacryloyloxyethyl phosphorylcholine via photo-induced self-initiated surface graft polymerization without additional photoinitiators. The modified PEEK surfaces exhibited exceptional wettability, protein-repellent properties, and improved tribological qualities [181,182]. Chouwatat et al. [183] utilized the formation of the self-induced radical moieties to graft electrolyte polymer brushes, i.e., cationic poly(2-(methacryloyloxy) ethyltrimethylammonium chloride) and anionic poly(3-sulfopropyl methacrylate potassium salt). As a result, the friction coefficient of the PEEK surface was reduced in wet conditions (Figure 9b). The study by Zheng et al. demonstrated self-grafted polyvinyl sulfonic acid sodium on PEEK surfaces and introduced sulfonate functional groups to the surfaces. These moieties increased surface hydrophilicity but did not alter surface topography. In addition, in vitro cell (MC3T3-E1 osteoblasts) adhesion, spreading, proliferation, and osteogenic differentiation were improved [184]. In the following study, polyvinyl phosphonic acid was grafted instead of polyvinyl sulfonic acid sodium, thus introducing phosphate functional groups to PEEK surfaces. Similar results as PEEK with sulfonate functional groups were observed. In vivo experiments in rabbits indicated improved bone-implant contact [185]. Yousau et al. evaluated self-surface *grafting to* and *grafting from* (assisted by UV-irradiation) using six different monomers and polymers, i.e., styrene, butyl acrylate, vinyl phosphonic acid (VPA), acrylic acid, polyacrylic acid (PAA), and monomethoxy terminated oligo (ethylene glycol) methacrylate (MeOEGMA). PEEK surfaces modified with PAA, and PVPA exhibited pH-responsive properties that shifted surface wettability. PEEK grafted with polyMeOEGMA was non-fouling against *E. coli* (Figure 9c) [186]. In addition, grafted PAA enhanced the wear resistance of the PEEK surface because PAA brushes could support high contact stresses (Figure 9d) [187].

A different route of surface photopolymerization was proposed by Liu et al. In this approach PEEK surfaces were coated with electrospun titanium dioxide (TiO_2_) nanofibers. Methacrylated hyaluronic acid (MeHA) was then deposited and polymerized using UV irradiation. Electrospun TiO_2_ provides surface roughness and a more favorable elastic modulus, while MeHA polymer backbone provides interaction sites for mesenchymal stem cells (MSCs). Biological characterizations showed increased cell adhesion, proliferation, and osteogenic differentiation [188].

### 4.4. Physical Surface Modifications of PEEK

**Figure 10:**
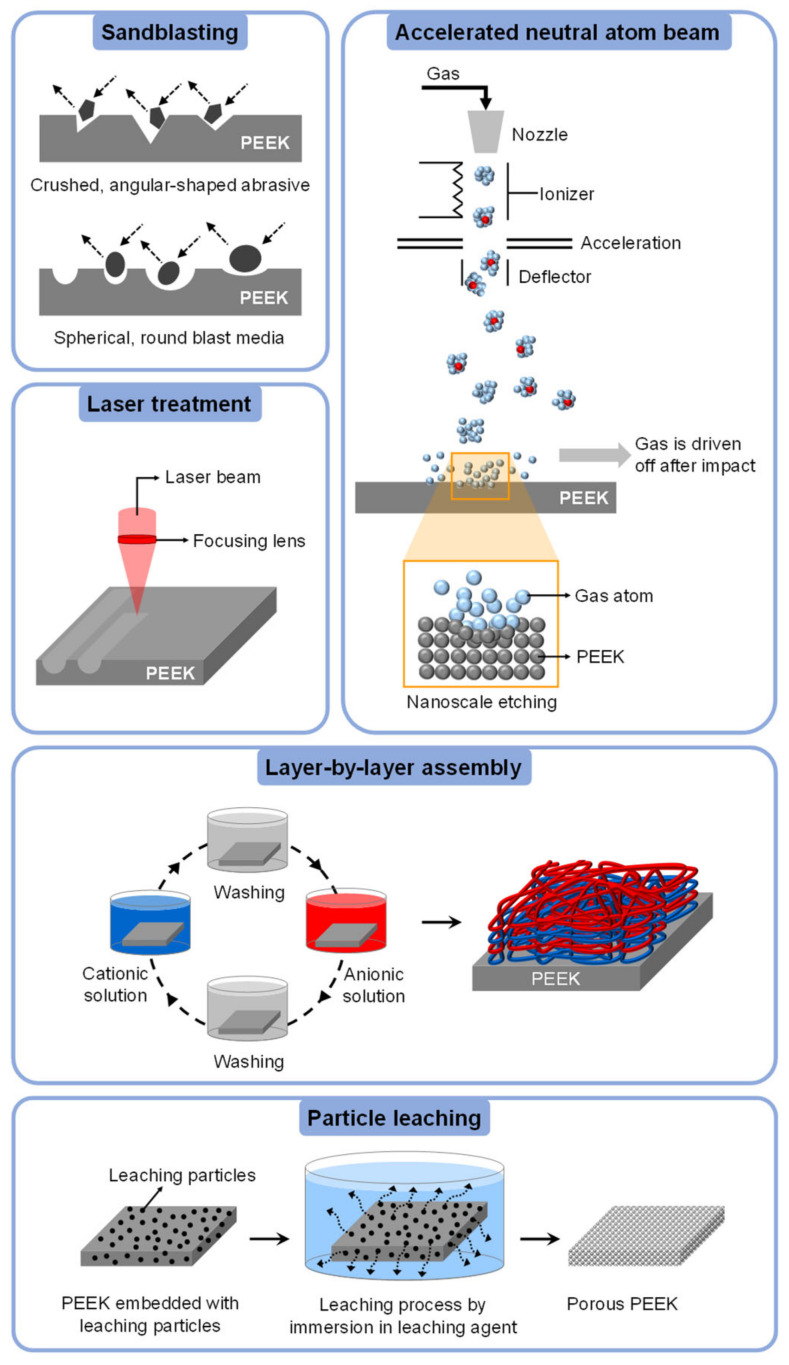
Illustration of physical surface modifications of PEEK. The figure of the accelerated neutral atom beam is inspired by previously published literature [189].

This section will highlight five physical surface modification techniques that are more frequently applied for altering the surface properties of PEEK, i.e., sandblasting, laser treatment, accelerated neutral atom beam (ANAB), layer-by-layer (LbL) assembly, and particle leaching. These methods are illustrated in Figure 10.

#### 4.4.1. Sandblasting

PEEK sandblasting is a surface roughening process using abrasive particles such as aluminum oxide (Al_2_O_3_), silicon oxide (SiO_2_), and titanium oxide (TiO_2_ [25,190] or via polishing against abrasive papers [191]. Nevertheless, the most common modification method is via sandblasting using Al_2_O_3_ particles [192]. The resulting surface roughness depends on the abrasive particle size [25,192] or the mesh size of the abrasive paper [191]. In addition, the sandblasting process duration and the sandblasting angle relative to the surface also affect the resulting surface topography [193]. Deng et al. sandblasted PEEK composites consisting of PEEK, carbon fibers, and nanohydroxyapatite (made of Ca(NO_3_)_2_⋅4H_2_O and (NH_4_)_2_HPO_4_) using Al_2_O_3_ particles with three grouped grain sizes, i.e., 60–80 µm, 110–150 µm, and 180–250 µm. A bigger particle size led to a rougher surface, i.e., 0.93 µm (lowest roughness), 1.96 µm (moderate roughness), and 2.95 µm (highest roughness), respectively, which proportionally enhanced surface hydrophilicity and calcium ion concentration on the surface. In vitro tests against osteoblast-like MG-63 cells indicate samples with moderate roughness displayed enhanced cell attachment, cell proliferation, ALP activity, and calcium nodule formation. In addition, in vivo osseointegration on moderately roughened surfaces was also showed improvement [192]. Sunarso et al. evaluated the performance of roughened PEEK surfaces (sandblasted by alumina particles, R_a_ = 2.3 µm) versus polished surfaces (polished by silicon carbide sandpaper, R_a_ = 0.06 µm). In vitro experiments with rBMSCs on sandblasted surfaces showed higher cell proliferation and differentiation, such as higher osteocalcin expression and bone-like nodule formation. In vivo, osseointegration was improved, as evident from pull-off force measurements (four times higher compared to that of the polished surfaces) [194].

Tang et al. compared abrasive paper (mesh size was 1200, 800, and 400) with abrasive particles to sandblast composite surfaces made from PEEK and nano calcium silicate. The results showed that surface roughness was increased with a decrease in mesh size: 1.06 μm for 1200 mesh, 1.13 μm for 800 mesh, and 1.48 μm for 400 mesh, while untreated composites had a roughness of 1.58 μm. On the contrary, when the composite surfaces were sandblasted with abrasive particles (note: the type of particle was not mentioned in the literature), the surface roughness was increased to 3.82 μm. The surface wettability of all sandblasted samples improved, with the water contact angle of particle-sandblasted surfaces being the lowest. Further, surface mineralization was also significantly enhanced for all treated surfaces. Biological characterizations demonstrated the highest promotion of osteoblast responses for composites treated with abrasive particles [191]. This study indicated that the performance of abrasive particles is superior compared to that of abrasive paper.

Some studies revealed that sandblasting with Al_2_O_3_ particles could improve the shear bond strength of PEEK surfaces [56,195,196]. Sandblasting with 50 μm Al_2_O_3_ particles at a pressure of 2.7 atm for 15 s, from10 mm perpendicular to the surface, increased the shear bond strength from ca. 5.58 MPa (untreated PEEK) to ca. 11.65 MPa [56]. A slightly lower shear bond strength was observed using bigger Al_2_O_3_ particles with a size of 110 μm (ca. 10.81 MPa). This effect might result from different experimental conditions (pressure = 2 atm, duration = 15 s, distance = 10 mm) [105]. In a recent study, the impact of sandblasting using alumina particles on PEEK adhesiveness was compared to that of silica-modified alumina particles. It was demonstrated that the shear bond strength of PEEK (reinforced with 20% ceramic) surfaces sandblasted with 30 μm silica-modified alumina was higher than those treated with 110 μm non-modified alumina. The shear bond strength values were ca. 24.1 MPa and ca. 15.2 MPa, respectively [197].

Nevertheless, contradictive studies showed that sandblasting had negligible effects on shear bond strength (SBS). Adem et al. showed that there was no significant difference in SBS between PEEK surfaces sandblasted with 50 μm Al_2_O_3_ particles (SBS = ca. 6.43 MPa) and untreated PEEK (SBS = ca. 5.39 MPa) [198]. Tosun et al. also concluded that surface sandblasting with Al_2_O_3_ particles or silica-coated Al_2_O_3_ particles did not lead to any changes in surface properties (i.e., surface roughness and SBS) aside from surface wettability [199].

#### 4.4.2. Laser Treatment

Laser treatment is a non-contact process to tailor surface geometry in a controlled manner [25]. The fabricated surface geometry ranges from irregular structures [106,200] to regular structures (e.g., channel, lattice, circle pore, etc.) [201,202,203]. The dimensions of the engraved pattern can be tuned by controlling the laser fluence and the number of pulses [201]. It was also reported that the laser wavelength used had an impact on tailoring the surface wettability [204].

**Figure 11:**
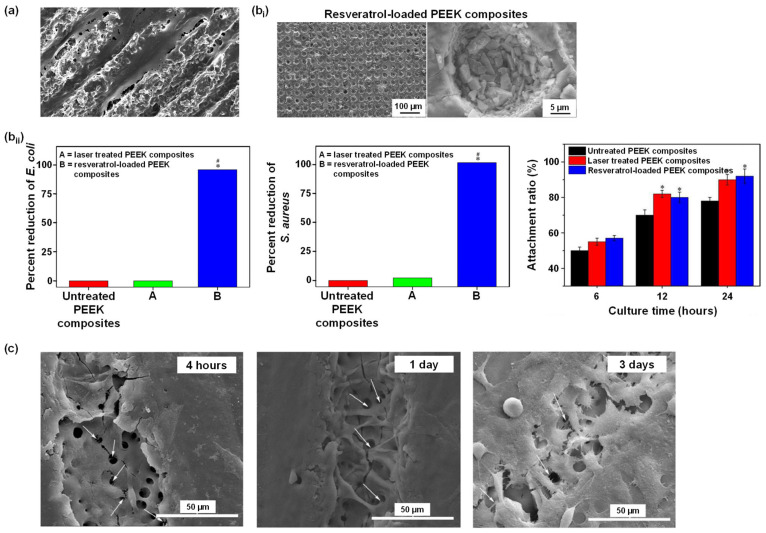
Laser treatment of PEEK surfaces. (**a**) Gheisarifar et al. observed cell alignment and elongation following the geometry of the surface topography (grooves) [40]. (**b**) The work from Cai et al.: (**b_i_**) SEM images showed porous PEEK composites loaded with antibacterial agent resveratrol; (**b_ii_**) antibacterial activity (* *p* < 0.05 against untreated PEEK, # *p* < 0.05 against laser treated PEEK) and cell attachment ratio (* *p* < 0.05 against untreated PEEK), respectively [205]. (**c**) SEM images showed the morphology of MC3T3-E1 pre-osteoblasts cultured for 4 h, 1 d, and 3 d. *White arrows*: cell pseudopodia protruding into the laser-generated pores [206]. All figures were re-printed with permission from the literature [40,205,206].

Gheisarifar et al. [40] investigated in vitro interaction between laser-grooved PEEK surfaces with the soft tissue of human gingival fibroblasts. Physical characterizations after laser treatment showed that surface roughness was increased, and while the surface chemistry composition was maintained, the surface wettability decreased. In vitro tests indicated that surface topography (grooves, the dimensions were not described in the literature) aided the cell alignment where the cells were elongated with more pseudopods attached to the grooves (Figure 11a). These results were supported by Cordero et al. Parallel groove pattern with ca. 40 μm in width was generated on PEEK surfaces using ArF excimer laser pulses (λ = 193 nm). The distance between the grooves was varied, i.e., 25, 50, 75, and 100 μm. The chemical composition of the surface was not affected by the laser treatment. In vitro, MC3T3-E1 pre-osteoblastic cells preferred to orient themselves following the groove pattern. In addition, the cell growth was most pronounced on 25 μm separated grooves [201].

A different surface pattern was evaluated by Huang et al. [203]. PEEK was reinforced with lamellar hydroxyapatite, and graphene oxide was etched using an ultra-short pulse laser. Three different pores with diameters of 200, 400, and 600 μm were generated. The depth of the pore was ca. 50 µm. The generation of these relatively large microstructures did not affect the mechanical properties of the composites. In vitro, cell (MC3T3-E1) proliferation on the laser-treated composites was enhanced compared to that on unmodified composites, especially on 400 µm pores. This cell viability was associated with the release of bioactive ions from hydroxyapatite which was more exposed after laser treatment. Much smaller pore dimensions were created using a femtosecond laser by Cai et al. [205]. Laser etching was done to the surface of PEEK-nanoporous magnesium calcium silicate composites, resulting in hierarchically porous surfaces. The particle diameter of the nanoporous magnesium calcium silicate was approximately 200 nm with a nanopore size of about 4 nm. The laser-generated micropores were about 20 μm in diameter. Interestingly, the laser treatment also generated sub-micropores at the internal surface with a diameter ca. as 0.5 μm. Thus, the laser-modified surfaces had a hierarchical micropattern. Following the laser etching, an antibacterial agent, resveratrol, was then loaded into the porous surfaces (Figure 11b_i_). A slow release of resveratrol was observed from the laser-treated surfaces. On the contrary, unmodified composites suffered from burst release of the resveratrol. It was shown that the use of resveratrol could inhibit the growth of *E. coli* and *S. aureus* (Figure 11b_ii_ left and middle). In vitro experiments were conducted using rBMSCs, and the results showed positive improvements: cell adhesion and proliferation were promoted, and osteogenic differentiation and bone-related gene expressions were enhanced (Figure 11b_ii_ right).

Combining laser treatment with other methods can bring synergistic effects; for example, Zheng et al. combined laser and plasma treatment methods to modify the PEEK surface. Laser treatment was conducted using CO_2_ laser, creating parallel grooves with 58 μm in width and 250 μm in distance. In addition, micropores with a diameter of ~4 μm were observed on the surface of the microgrooves. Plasma treatment was conducted to modify the surface chemistry by introducing carboxyl functional groups. For this, acrylic acid was polymerized on the PEEK surfaces using the plasma surface polymerization technique. This plasma treatment did not change the surface morphology. Moreover, the mechanical properties were unaltered after the dual treatment. Biological performance was characterized in vitro using MC3T3-E1 pre-osteoblasts showing cell preference to adhere, spread, and proliferate on the dual-treated surfaces compared to single-treated surfaces. In addition, cell pseudopodia protruded into the laser-generated pores, indicating bone-implant integration (Figure 11c) [206]. Further, when nitrogen plasma treatment was carried out on the laser-grooved PEEK surfaces, as mentioned above [40], the proliferation of human gingival fibroblasts significantly showed improvement (compared to that on laser-only-treated surfaces). Thus, the plasma treatment showed a synergistic interaction with laser treatment [40].

The effect of laser treatment on the adhesiveness of PEEK is still unclear [162]. Pulsed ytterbium laser (Yb:PL) treatment on PEEK surfaces could increase shear bond strength from ca. 5.09 MPa (untreated PEEK) to ca. 11.46 MPa [105]. Neodymium-doped yttrium orthovanadate (Nd:YVO_4_) laser treatment improved the shear bond strength between PEEK and resin-based luting agents, where the improvement depended on the penetration depth of the laser, i.e., a more profound depth led to a higher shear bond strength. In this case, the Nd:YVO_4_ laser was used to fabricate grooves in lattice pattern with an interval of approximately 200 µm, and groove depth of 100, 150, and 200 µm. The mean shear bond strength of untreated PEEK to four different luting agents was ca. 4.5 MPa, while those of modified PEEK were ca. 13.2 MPa (100 µm groove depth), ca. 14.4 MPa (150 µm groove depth), and ca. 15 MPa (200 µm groove depth) [202]. Ulgey et al. compared the resulted shear bond strength between PEEK and veneering materials after treatment with neodymium-doped yttrium aluminum garnet (Nd:YAG) laser, erbium-doped yttrium aluminum garnet (Er:YAG) laser, and potassium titanyl phosphate (KTP) laser. The laser treatments generated irregular structures on the surfaces and increased the shear bond strength, i.e., ca. 11.33 MPa after KTP laser modification, ca. 14.29 MPa after Er:YAG laser treatment, and ca. 16.35 MPa after Nd:YAG laser treatment, while the untreated PEEK adhesiveness was ca. 8.09 MPa. Hence, this work indicated that Nd:YAG laser treatment was the most effective method [207]. Another study reported that CO_2_ and Er:YAG lasers increased the shear bond strength of PEEK to veneering resin from ca. 7.7 MPa (untreated PEEK) to ca. 10.6 MPa (CO_2_ laser-treated PEEK) and to ca. 14.4 MPa (Er:YAG laser-treated PEEK) [208]. Combining Er:YAG laser treatment with sandblasting method could improve the adhesive properties even further. Shear bond strength as high as ca. 22 MPa was achieved by Taha et al. by combining Er:YAG laser and sandblasting treatments [209].

However, work by Ates et al. demonstrated that Er:YAG laser treatment had no positive effect on bonding strength between PEEK and veneering resin. The irregularly laser-structured PEEK had approximately 6.03 MPa of shear bond strength, which was similar to that of untreated PEEK (ca. 6.35 MPa). Combining Er:YAG laser treatment with sandblasting using 50 μm Al_2_O_3_ particles improved the adhesiveness to ca. 12.09 MPa. Improved results were achieved by pairing Er:YAG laser treatment with silica coating treatment, which increased the shear bond strength to 13.14 MPa [106]. Caglar et al. [56] also discovered that Er:YAG laser treatment did not improve the adhesiveness of PEEK. Another negative result was reported by Henriques et al., where a CO_2_ laser (λ = 1064 nm) was employed to fabricate pores (diameter = 200 μm, the distance between pores = 400 or 600 μm) on the surface of PEEK and its composites. The surface laser modification failed to improve the bonding strength between PEEK and resin cement, as evident from the shear bond strength measurements. SEM images further showed the inability of resin cement to flow into the laser-created pores. Filling the pores with cement is crucial to establish mechanical interlocking, without which the bonding strength will suffer [210].

**Figure 12:**
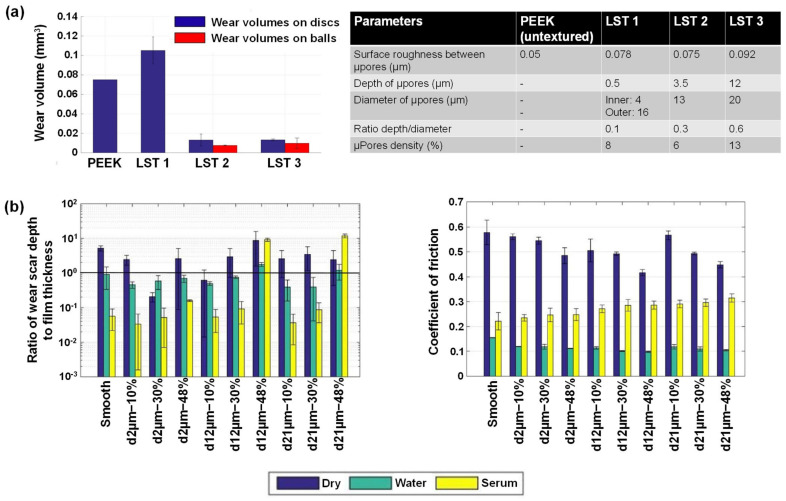
The effect of laser treatment on tribology-resistance. (**a**) Hammouti et al. showed a reduction in wear rate for micropatterned (micropores) PEEK surfaces [211]. (**b**) Dufils et al. demonstrated reduced wear and friction on microstructured PEEK surfaces (micropores coated with DLC film), especially those with shallower micropores and lower area coverage (in water and serum, respectively) in comparison to untreated PEEK [214]. All figures were reprinted with permission from the literature [211,214].

Only limited research studies were conducted to understand the effect of laser treatment on the tribological behavior of PEEK. Hammouti et al. used a femtosecond laser to etch the PEEK surface to generate micropores with a diameter ranging from 13–20 μm, spacing ca. 50 μm, up to a depth of ~0.5–12 μm. The tribological performance was measured using a uni-directional ball-on-disc configuration with bovine calf serum as a lubricant (loading force = 5 N, sliding velocity = 0.001 m/s). Compared to flat PEEK, the structured PEEK showed a higher friction coefficient and reduced wear rate (ten times lower). It was also revealed that a higher depth of micropores led to a higher friction coefficient but a higher wear resistance (Figure 12a) [211]. In another work, tribological experiment was conducted on PEEK surface bearing micropores with diameter of 25 μm (depth = 6 μm, spacing = 266 μm) and 50 μm (depth = 80 μm, spacing = 136 μm). The micropores were generated using a picosecond laser, and the tribological properties were assessed on a ball-on-flat configuration under dry conditions (loading force = 0.9 and 3 N). Superior friction and wear properties were identified on PEEK substrates with 50 µm micropores [212]. Wyatt et al. utilized pin-on-disc tribometer to assess friction coefficient of PEEK reinforced with carbon fiber. Prior to the tribological tests, the surface of PEEK composites was structured using laser treatment, resulting in micropores with a varied diameter (ca. 50–130 μm), spacing (ca. 50–335 μm), depth (ca. 16–52 μm), and area coverage (ca. 6–20%). All structured surfaces were found to have reduced friction coefficient compared to flat PEEK composites. However, data showed that the tribological performance varied depending on the micropore dimensions. Thus, it was concluded that further studies are necessary to find the optimal surface topography for enhanced tribological performance. The work of Wyatt et al. found that 150 µm (in diameter) micropores, with a spacing of 175–200 μm and area coverage of 10–15%, gave the best result when the tribological tests were carried out in saline lubrication (contact pressure = 2 MPa, sliding speed = 50 mm/s) [213]. Dufils et al. combined laser treatment with diamond-like carbon (DLC) film deposition to reduce PEEK wear. PEEK surfaces were exposed to ultrashort-pulse laser etching, creating micropores with diameter of about 30 µm, and varying depth (2, 12, and 21 μm) and area coverage (10, 30, and 48%). DLC film was then deposited onto the structured PEEK surfaces. Surface characterizations revealed deeper micropores, and higher coverage areas led to surface deformation and DLC film cracking. Tribological properties were evaluated by rubbing the modified PEEK surfaces against an alumina ball in dry condition, water, and bovine calf serum. The results showed shallower micropores and lower area coverage could significantly reduce friction and wear in water and reduced wear in bovine calf serum compared to control samples (flat DLC-coated PEEK) (Figure 12b) [214].

#### 4.4.3. Accelerated Neutral Atom Beam (ANAB)

ANAB is a surface treatment where the surface is bombarded with energetic neutral atoms produced by a gas cluster ion beam [215]. As a result, a surface with nanoscale structures is obtained. Khoury et al. exposed PEEK substrates to argon ANAB, and surface characterizations showed improved hydrophilicity from ca. 92° to ca. 73° and a nanostructured surface (however, surface roughness was decreased from R_a_ = 4.63 nm to R_a_ = 3.45 nm). The bioactivity was assessed using dental pulp stem cells where their attachment and proliferation were increased. In addition, accelerated osteogenic differentiation was also observed [216]. ANAB-treated PEEK (argon gas treatment) also promoted the proliferation of human osteoblast-like cells, increased the osteogenic expression (alkaline phosphatase 1.98-fold, runt-related transcription factor 2 3.2-fold, collagen1A1 1.94-fold, integrin-binding sialoprotein 2.78-fold, and bone morphogenetic protein 2 1.89–fold), and enhanced mineralization up to 6.4-fold at 21 days. In vivo studies using sheep showed that bone-implant contact was improved by 3.09 fold, and push-out strength increased by 2.07 fold, leading to bone ingrowth (both at early (4 weeks) and later stages (12 weeks)) [217]. Another in vitro study using human mesenchymal stem cells (hMSCs), human osteoblasts (hOB), and skin fibroblasts (BR3G) also demonstrated cell growth on PEEK modified with ANAB (argon gas) was superior compared to unmodified PEEK [218]. Remarkable results were reported by Webster et al., which showed ANAB-modified PEEK exhibits both enhanced cytocompatibility and antibacterial properties without the addition of antibacterial agents. In vitro, osteoblast response on ANAB-treated PEEK was improved in terms of increased deposition of calcium-containing minerals, ALP activity, and osteocalcin, osteopontin, and osteonectin synthesis, compared to those on unmodified PEEK. Furthermore, the ANAB treatment increased the absorption of proteins such as mucin, casein, and lubricin onto the PEEK surfaces, reducing *S. aureus*, *E. coli*, and *S. epidermis* bacteria adhesion [219].

#### 4.4.4. Layer-by-Layer (LbL) Assembly

LbL technique employs the deposition of oppositely charged polymers (polycations and polyanions) on a charged surface via electrostatic interactions [220]. Other forces that facilitate LbL film deposition include hydrophobic interaction, covalent bonding, host-guest interaction, and hydrogen bonding [221,222,223]. This method provides certain advantages, such as precise control of film thickness and its properties, low-cost production, the possibility to incorporate and deliver bioactive molecules in a controlled manner, etc. [224]. Unfortunately, literature discussing the utilization of LbL assembly to modify PEEK surface is limited. In the scope of this review article, only physical LbL formation via electrostatic interaction will be discussed.

**Figure 13:**
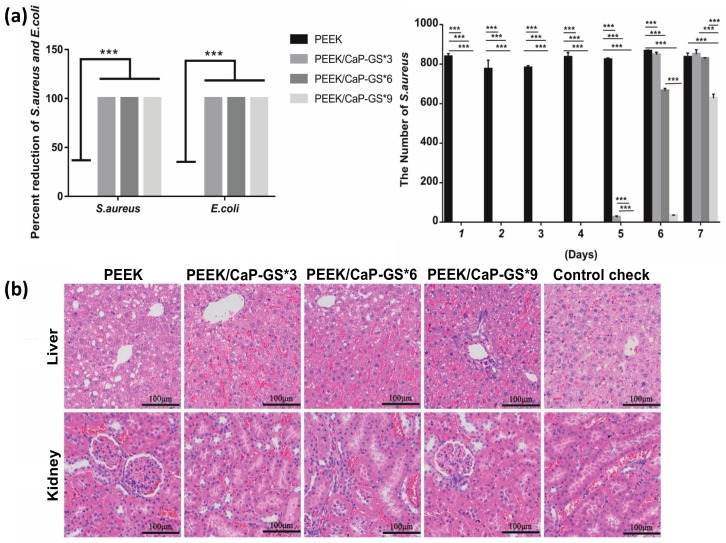
PEEK surface modification using layer-by-layer technique. Xue et al. showed (**a**) antibacterial properties against *S. aureus* and *E. coli* and (**b**) in vivo histological analysis (scale bar: 100 µm) of LbL-modified PEEK surfaces. PEEK = untreated PEEK. PEEK/CaP-GS*n (*n* = 3, 6, 9) = PEEK layered with *n* layers of brushite containing antibiotic gentamicin sulfate. Control check = blank [154]. LbL-(brushite-gentamicin)-modified PEEK surfaces enhanced antibacterial and osseointegration properties. (*** The differences were considered statistically significant at *p* < 0.001). All figures were reprinted with permission from the literature [154].

Xue et al. used LbL method to modify PEEK surface. First, the PEEK surfaces were activated by immersion in poly(ethylenimine) solution followed by immersion in poly(styrene sulfonate) solution. The activated PEEK was immersed in Ca(NO_3_)_2_·4(H_2_O), followed by immersion in gentamicin sulfate-containing (NH_4_)_2_·HPO_4_ to assemble Ca^2+^ ions and antibiotic gentamicin sulfate, respectively. The deposition of Ca^2+^ ions and gentamicin sulfate layer were repeated 3, 6, and 9 times to obtain different layer thicknesses of composite containing Ca^2+^ ions and gentamicin sulfate on the activated PEEK. From this LbL process, PEEK surfaces functionalized with brushite (CaHPO_4_.2H_2_O) layers containing antibiotic were obtained. Both in vitro (using *S. aureus* and *E. coli*) and in vivo (in rats) experiments showed that the brushite-gentamicin-modified PEEK surfaces exhibited antibacterial and osseointegration properties (Figure 13a,b, respectively). PEEK bearing 6 LbL layers was found to present the best antibacterial and osseointegration performance [154].

Liu et al. assembled nanoscale 5 to 20 layers of polyanion poly(allylamine hydrochloride) and polycation poly(styrene sulfonate) on PEEK surfaces. Cell activity was assessed in vitro using bone marrow stromal cells, and the results showed that cell adhesion, cell proliferation, cell growth rate, and ALP activity were significantly improved. Animal studies using a rabbit model and 20 LbL layer PEEK demonstrated improved osseointegration. In addition, samples with 20 LbL layers also presented the lowest water contact angle [225].

Deng et al. combined sulfonation and LbL techniques to incorporate antibacterial properties and enhance biocompatibility of PEEK. The LbL multilayers consisting of Zn ion-containing chitosan (as positively charged polyelectrolyte layer) and Ag ion-containing sodium alginate (as negatively charged polyelectrolyte layer) were deposited onto sulfonated PEEK. Antibacterial assay was conducted against *E. coli* and *S. aureus*, and the results showed that the presence of Ag ions effectively inhibited the growth of the tested bacteria. Surface biocompatibility was also improved due to the presence of porous structures (from sulfonation process) and Zn ions, which included an enhanced cell viability, spreading, and proliferation, as well as accelerated osteo-differentiation, and osteo-maturation [226].

#### 4.4.5. Particle Leaching

Particle leaching is a method to introduce porosity on a surface by leaching the particles embedded either in bulk or on the surface of a material. Common particles include NaCl salt, sucrose, etc., with NaCl as the most used particle. Leaching is executed by solving the particles in respective solvent, leaving behind a porous surface [227]. Torstrick et al. demonstrated that pore dimensions can be tailored by adjusting the diameter of NaCl particles [228]. Santos et al. deposited NaCl layer into PEEK surfaces using hydraulic press operating at 850 kg/cm^2^ and 390 °C for 20 min. After cooling, the substrates were immersed in water for 72 h (room temperature), followed by immersion in stirred boiling water for 1 h. The produced pores were uniform with a defined pore interconnectivity and an average diameter of ca. 270 µm. The mechanical properties of PEEK after NaCl leaching were, unfortunately, decreased. Biological characterization using fibroblast cell line resulted in 86% of cell viability [227]. Evans et al. leached incorporated NaCl particles from PEEK, and the generated porous PEEK maintained 73.9% of its original strength, 73.4% of its elastic modulus, and 73.4% of its fatigue resistance. Osseointegration in vivo was shown to be improved compared to that of non-porous PEEK [117]. In the follow-up study, three groups of NaCl with different diameters (200–312, 312–425, 425–508 µm) were leached from PEEK, leaving porous surfaces with porosity of ca. 60–70% and pore interconnectivity of >99%. Mechanical tests showed that the porous PEEK exhibited > 50% decrease in ductility compared to nonporous PEEK. However, all analyzed porous substrates were favourable for cell growth, independent of the pore size [228]. A rather different approach was executed by Yuan et al. where hydroxyapatite microspheres were used as the leaching particles and hydrochloric acid was used as the leaching agent. Moreover, the particle leaching was combined with sulfonation. In short, PEEK-hydroxyapatite composites were immersed in 37% HCl to remove the hydroxyapatite particles. The resulting porous PEEK was further immersed in 80% sulfuric acid, followed by immersion in simulated body fluid. The particle leaching process provided interconnected macropores, the sulfonation induced formation of nano- and micropores, while the simulated body fluid deposited bone-like apatite. The particle leaching led to surface porosity of 77.4% and the subsequent sulfonation-simulated body fluid treatment reduced the surface porosity to 70.7%. The final pore diameter was about 160 µm. In vivo, remarkable enhanced osseointegration was detected, as confirmed by bone growth into the pores. On the contrary, only a handful of bone growth was observed on the unmodified surfaces. In addition, the new bone push-out force was also improved, indicating a better osseointegration on modified surfaces than that on unmodified surfaces [116].

## 5. Conclusions

In dentistry, polyaryletherketones (PAEKs) are a class of high-performance polymers employed for numerous clinical cases. This class includes numerous members with various chemical structures, among which PEEK is the most widely used in dentistry due to its similar bulk properties as bone. Our review addresses various aspects of surface modification and treatment methods to generate PEEK as a desirable alternative to metal- and ceramic-based dental materials. The surface-related properties such as adhesiveness to other materials, osseointegration, antibacterial, and tribological (friction-wear) are critical and uncompromisable in a material when employed in dentistry. Hence, various physical and chemical surface treatment processes, such as air abrasion, laser treatment, sulfonation, plasma treatment, etc., present effective approaches to address the requirements. Literature showed that the surface treatment approach could simultaneously impact several properties, both in desired or undesired ways; hence a careful optimization of surface parameters or combinatorial modification approaches are often required.

This review suggested that highly durable and resilient polymeric adhesives, liners, and conditioners, when added to the PEEK (pre-treated) surface, successfully bond PEEK with tissue, veneer materials, or titanium implant bases. To this end, a section in this review discusses the strategies for improving the performance of PEEK using acrylic- and silicon-based polymers. In addition, the review presented that surface roughness, controlled via various surface treatment approaches, is a critical parameter that can enhance PEEK adhesion to other materials via enhanced surface energy and/or mechanical interlocking. Both in vitro and in vivo studies showed this approach to be effective in enhancing the interactions with tissue cells. Nevertheless, higher surface roughnesses (R_a_ > 0.2 μm) can also assist bacterial adhesion and biofilm formation, which is shown to be overcome by incorporating various antibacterial compounds into the PEEK surface. Furthermore, various growth factors and cell-attractive biomolecules attached to the PEEK interface are shown to enhance the desired cell growth and proliferation.

The combined strategies of controlling the surface topography, along with the incorporation of adhesive polymers, antibacterial compounds, and osteogenic compounds have resulted in the enhanced biological performance of PEEK for various applications in dentistry. The high performance of PEEK polymers for dentistry was quantified from surface roughness, porosity, shear bond strength, in vitro experiments using various bacterial strains and cell lines, in vivo experiments using model animals, and tribological properties. We conclude that both surface chemistry (functional groups) and surface physical topography (porosity, roughness) play a critical role in influencing the attachment and bioactivity of PEEK to other materials, whether it is biological entities like bacteria and tissue cells or other materials like titanium, veneer composite, adhesive cement, etc. In general, the presence of PEEK on dental materials like titanium (either surface-coated as a veneer or blended as a multi-material) helps in reducing frictional wear in artificial saliva, hence presenting fewer health concerns from debris ingestions. This review sheds light on the design rules of high-performance polymers, especially PEEK, in dentistry.

## Data Availability

Not applicable.
